# Health-Related Quality of Life of Patients with *Epidermolysis bullosa* and Carers Using a Time Trade-Off Approach in the UK General Population to Elicit Utilities for Health Technology Assessment

**DOI:** 10.3390/jmahp14030048

**Published:** 2026-08-11

**Authors:** Luke Stainer, George Morgan, Thomas Snell, Claire Mather, Sagair Hussain, Keith Tolley

**Affiliations:** 1 Tolley Ltd., Buxton SK17 6EQ, UK; luke@tolleyhta.com (L.S.); tom@tolleyhta.com (T.S.); 2 HCD Economics, The Innovation Centre, Keckwick Ln, Daresbury, Warrington WA4 4FS, UK; george.morgan@dht.health; 3 DEBRA UK, The Capitol Building, Oldbury, Bracknell RG12 8FZ, UK; claire.mather@debra.org.uk (C.M.); sagair.hussain@debra.org.uk (S.H.)

**Keywords:** *Epidermolysis bullosa*, patient utility, carer utility, rare disease, time trade-off, health technology assessment

## Abstract

Background: Epidermolysis bullosa (EB) is a complex group of rare, inherited skin disorders that blister easily, imposing substantial burden on patients and carers. There is a lack of published evidence in the UK quantifying health-related quality of life in EB patients and carers as utility values suitable for use in Health Technology Assessment (HTA). Methods: A time trade-off (TTO) study was conducted with 120 UK general public members to estimate utility values for EB patient and primary carer vignettes depicting varying EB severity. An exploratory sub-study involving six carer and clinical experts was conducted to estimate utility values of second carers. Results: 115 general public participants were included in the final TTO analysis. Mean utility values declined as disease severity increased, from 0.82 in the least severe state, to 0.54 in the most severe. Carer utilities were higher than the equivalent patient health states, but with a similar pattern of decreasing utility with increasing EB severity, ranging from 0.85 to 0.64. The secondary carer sub-study, in six respondents estimated a second care burden factor relative to the primary carer of 77%. Conclusions: The utilities estimated for both EB patients and their carers have relevance for future economic evaluations and HTA of new EB therapies.

## 1. Introduction

Epidermolysis bullosa (EB) is a group of complex, rare, inherited skin disorders that cause the skin to be extremely fragile and blister easily. EB is caused by more than 1000 known mutations in at least 21 genes encoding anchoring proteins of the dermal-epidermal junction, which help maintain skin integrity [1,2]. Based on revised EB consensus classifications, EB is categorised into four major EB types according to the level of skin cleavage present and can be inherited in a dominant or recessive manner: EB simplex (EBS); Junctional EB (JEB); Dystrophic EB (DEB), and Kindler EB (KEB) [3]. DEB and JEB are the most severe EB types, disrupting skin physiology to the deepest layers of the skin, and are the focus of this study.

Patients with DEB and JEB experience significant wound-related challenges, including a high wound burden, prolonged healing times, and a recurring cycle of wound breakdown. DEB and JEB are associated with debilitating symptoms such as anaemia, pain and pruritus (itching), as well as additional systemic complications including infections/sepsis, syndactyly, squamous cell carcinoma, failure to thrive, and oesophageal strictures [4,5]. The symptomatic burden of DEB and JEB can carry considerable morbidity, as well as an increased mortality risk, thereby having a substantial effect on the health-related quality of life (HRQoL) of both the patients and those that care for them [6].

There is no curative treatment for DEB or JEB. Current management focuses on wound management with frequent dressing and bandage changes, reducing potential for new injury, minimising complications, and improving quality of life [7,8,9,10].

Wound management can be one of the most challenging aspects of EB for patients with a high wound burden, and the informal carers who assist them (usually parents or siblings), as daily bathing, blister lancing/draining, and dressing changes can be extremely time-consuming (up to four hours per day), painful, and anxiety-provoking for parents caring for young children [11,12].

Until recently, there were no licenced drug treatments available for the management of EB. In 2022, birch bark extract (Filsuvez^®^) was approved as the first therapy for treating partial thickness wounds associated with DEB and JEB in patients 6 months and older, with recommendations for use from the United Kingdon (UK) health technology assessment bodies (HTA), the National Institute for Health and Care Excellence (NICE) and the Scottish Medicines Consortium (SMC) [13,14,15]. There is a gene therapy, beremagene geperpavec (B-VEC), currently undergoing appraisal at NICE for treating skin wounds associated with DEB, with guidance expected to be published in July 2026 [16]. There are several other pharmacological treatments in various stages of clinical development for EB; hence, over the next few years further technology appraisals can be expected in the UK to assess the clinical and cost-effectiveness of new therapies to determine reimbursement, each requiring evidence of HRQoL benefits and cost-effectiveness.

In the UK and other cost-effectiveness markets (e.g., Europe, North America and Australia), HTA bodies generally require patient HRQoL outcomes to be measured as utilities for inclusion in quality-adjusted life years (QALY) assessments of new therapies [17]. However, published utility evidence in EB remains limited; while some utility values have been derived from generic preference-based measures such as the EQ-5D, there is a particular lack of utility values stratified by EB disease severity for use in HTA and economic evaluations of EB therapies. The primary objective of this study was therefore to quantify the utility impact of DEB/JEB on patients and their primary informal carer, for different levels of EB severity. Additionally, the impact of disease on secondary carers, other than the primary carer, is a topic of interest in recent HTAs conducted by NICE [13]. To address this, a supplementary sub-study was carried out alongside the main utility study in family carers of patients with EB and clinical experts (proxy respondents), to quantify the relative burden and utility impact on secondary carers of people living with EB (e.g., other parent or family member). The utility values derived for patients, primary carers, and secondary carers were used to inform health state valuations in an EB cost-effectiveness model developed for UK HTA purposes [13].

## 2. Methods

### 2.1. Study Design

The utility study was conducted in the UK general public, designed to elicit utility values for six EB patient health states of varying severity, and three EB primary carer-related health states. Utility values were elicited from the general public to reflect a societal perspective, which is the preferred approach by UK HTA bodies for utility assessment [17]. A study plan was developed to detail the development of validated health state descriptions (vignettes), design of a time trade-off (TTO) data collection tool, and the conduct of the main utility elicitation study. A supplementary sub-study was designed to evaluate the relative burden and utility for a secondary carer (e.g., other parent, sibling, grandparent). Utility values were elicited using the TTO approach, whereby participants traded time spent in full health against a fixed, longer duration spent in poorer health states, to assess their relative preference and hence utility for each health state. The study design and methods employed in this utility study are consistent with established approaches [17,18,19,20].

### 2.2. Development of Validated Vignettes

The patient-relevant vignettes were aligned with the health states in a cost-effectiveness model developed for a new EB therapy (birch bark extract). The health states were based on a surrogate marker for EB severity, the Body Surface Area Percentage (BSAP) covered by wounds. The percentage ranges for BSAP health states (HS) in the economic model were as follows: HS1: 0–4%, HS2: 5–7%, HS3: 8–10%, HS4: 11–18%, HS5: 19–24%, HS6: 25%+, which were informed and validated by expert clinical opinion. Qualitative patient vignettes describing the HRQoL impact of living with DEB and JEB were therefore developed to align with the six BSAP severity health states. In addition, primary informal carer HRQoL vignettes were created for three of these health states (HS2, HS4, HS6; labelled HS2-CG1, HS4-CG2, HS6-CG3, respectively) to capture the informal carer impact. Carer vignettes were not developed for all six health states to minimise participant burden and fatigue; instead, for the purposes of the economic model, the carer HRQoL impacts estimated for HS2, HS4 and HS6 were assumed to apply also to HS1, HS3 and HS5, respectively. This assumption was considered clinically reasonable, as the BSAP ranges within each pair of health states are adjacent and sufficiently similar in severity when considering carer burden, and was validated by the EB clinical experts involved in the study, who confirmed that the carer burden profile would not differ meaningfully between paired states. A total of nine vignettes were therefore developed in the study.

The content of the vignettes was informed by studies identified through a targeted literature review (TLR) focused on the HRQoL burden associated with EB in children and adults, and also by clinical and patient expert input and review. The TLR was based on a previously published systematic literature review (SLR) (Togo et al. 2020) covering the HRQoL in people with EB [21]. The review from Togo was last updated in 2019, so the specific search strategy from this review was used to identify studies up to the end of 2020. Searches were conducted on 4 January 2021; the TLR extended the search to cover studies published between 16 May 2019 and 31 December 2020 using the same search strategy. Searches of electronic databases included MEDLINE (through the PubMed interface) and Cochrane Library, and relevant abstracts from the EB 2020 world conference. In addition, the condition-specific IscorEB questionnaire played a key role in the wording of the vignettes; it was designed to reflect daily-life impacts and HRQoL domains affected by EB, while remaining concise and clinically relevant [22]. They were refined through an iterative process to ensure both comprehensibility and clinical accuracy, with attention paid to minimising framing or labelling bias and participant fatigue. Patient and clinical expert input was incorporated throughout the vignette development process. Initial vignette content was informed by the targeted literature review and the IScorEB questionnaire, with iterative refinement guided by a steering group that comprised EB health researchers and economic modelling experts. Subsequently, three EB consultant dermatologists, all with extensive experience managing DEB and JEB, and an EB patient representative (a member of the patient body DEBRA UK), reviewed the vignettes for clinical accuracy and comprehensibility. During health state development, the experts reviewed the draft vignettes and accompanying wound diagrams and provided feedback on the clinical accuracy, completeness and clarity of the descriptions. The health states underwent multiple rounds of iterative review and refinement, with revisions made following each round of expert feedback until the final versions were agreed for pilot testing. This included feedback through one-to-one discussions, group review sessions and email correspondence. Feedback from this review led to refinements to the wording of symptom descriptions, clarification of wound management burden, and adjustment of severity descriptors, i.e., linking BSAP to IscorEB domains, to better reflect clinical practice. This iterative process ensured the final vignettes accurately represented the lived experience of DEB and JEB across the severity spectrum.

Following expert validation, a pilot study was conducted with 10 members of the UK general public to assess the clarity and comprehensibility of the health state descriptions, participants’ understanding of the cTTO methodology, the feasibility of the interview procedures and quality control of the elicited data. Participants reviewed and valued all health states. All participants reported that the language and content of the health state descriptions were clear and coherent. The pilot was designed to evaluate clarity and feasibility rather than formally assess discrimination between individual health states. All pilot participants reported that the language and content were clear and coherent. The finalised six patient vignettes and three carer vignettes are provided in Appendix A. The vignettes had two components: an anchor, which was consistent across all vignettes, representing a typical description of EB and its current management, and a severity-linked component which described the HRQoL and daily living impact associated with each level of EB severity (as proxied by BSAP). Each vignette described EB severity impact according to three dimensions: wounds and other symptoms, disease management, and impact on life. Patient vignettes were developed without explicit differentiation by patient age. During the vignette development process, EB clinical experts noted that disease impact likely differs depending on the patient’s age, with children and adolescents potentially being affected and managed differently within the healthcare system compared to adults. However, given that the study design involved valuation by adults from the general public, it was considered impractical for respondents to accurately imagine and assess child-specific health states, particularly for very young ages. There was also a concern that age-related bias could influence valuations, with respondents potentially assigning systematically higher utility values to child health states due to a preference for preserving life-years in younger patients. As such, no differentiation was made between health states by patient age, and wording related to the impact on daily life was described in sufficiently general terms to be applicable across age groups. Each vignette was accompanied by a diagram (based on Lund and Browder BSAP diagrams) [23] to help participants comprehend the level of wound coverage associated with each BSAP category. These diagrams depict both adult and child body figures, as is standard for this tool; however, they were included solely to illustrate the proportion and distribution of body surface area affected by wounds, and were not intended to imply that the vignettes described either a paediatric or adult patient specifically.

**Figure 1:**
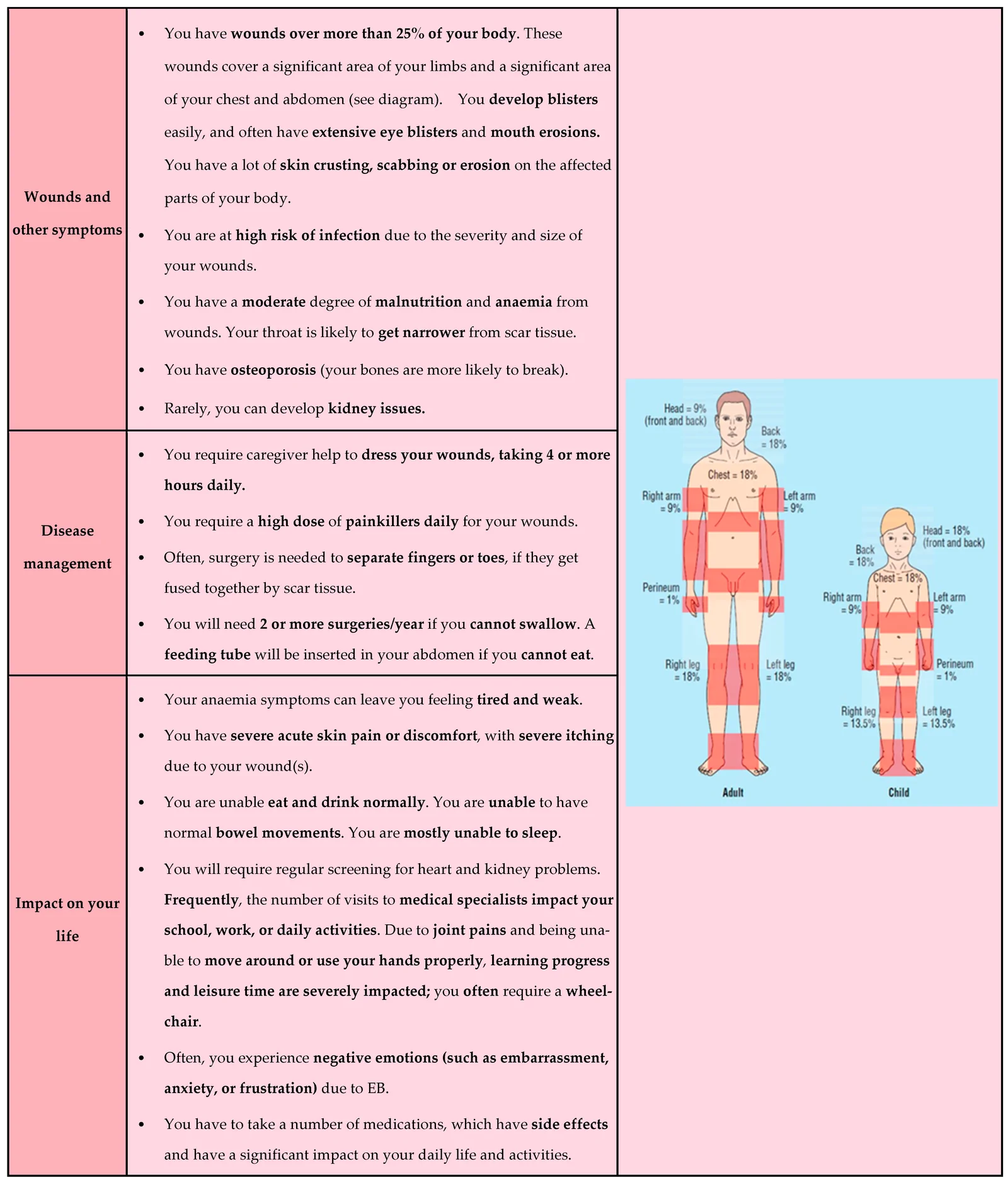
Health state 6 (patient-perspective) vignette with BSAP ≥ 25% of the body. Anatomical figure source—Lund and Browder [23].

**Figure 2:**
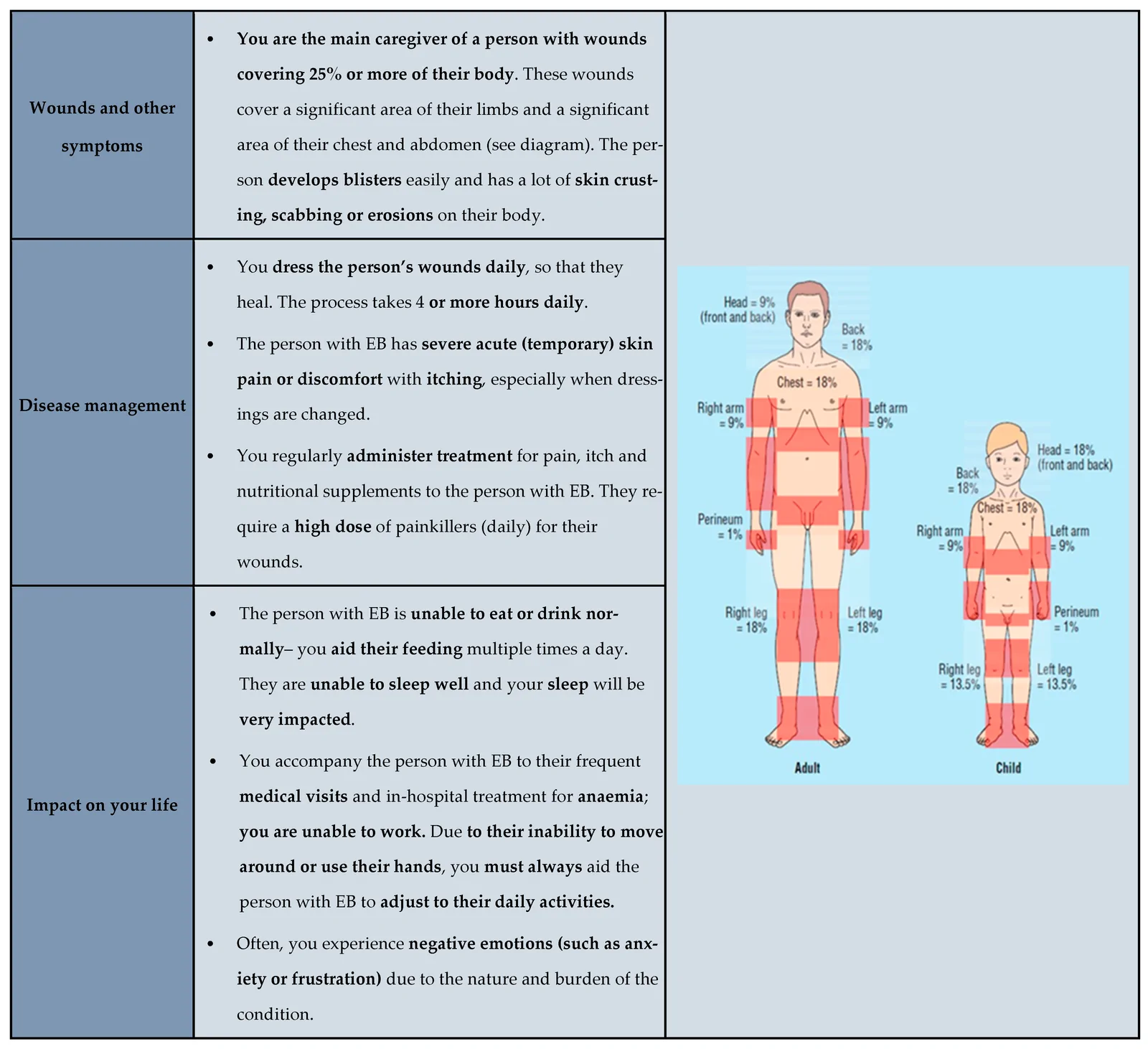
Health state 6 (carer perspective) vignette with BSAP ≥ 25% of the body. Anatomical figure source—Lund and Browder [23].

The three carer vignettes were valued from the perspective of a primary informal carer who provides (unpaid) care to a patient in the described health state. The wording for the three vignettes capturing carer-perspective impact was amended accordingly to permit appropriate valuation, i.e., “You are the main caregiver of a person with wounds covering…”. To ensure appropriate valuation of the carer health states, participants were explicitly instructed to consider the health state from the perspective of the described primary informal carer, focusing on the impact of caregiving on their own HRQoL, rather than directly on the patient’s symptoms. The patient symptom descriptions included in the carer vignettes were provided as contextual information to help participants understand the caregiving demands involved, rather than as states to be valued directly. The upper anchor for the carer TTO task was defined as a state in which the respondent is in full health and is not required to provide informal care, thereby capturing the full disutility associated with the caregiving role. This distinction was made explicit to participants during the interviewer-administered TTO task. As an illustration, Figure 1 and Figure 2 represent the patient and carer vignettes, respectively, for HS6. All patient and carer vignettes are presented in Appendix A.

### 2.3. Participants

Adults (≥18 years) from the UK general public who provided informed consent and demonstrated the ability to understand and respond to the study questions during screening were recruited. Participants were recruited via trained moderators making home door-to-door visits in a range of geographical areas in the UK until a sufficient quota of adult participants for the study was reached. Quotas were applied for age, gender, and geographical location to reflect the UK population, based on UK ONS estimates [24]. Age quotas were distributed across five categories (18–25, 26–40, 41–55, 56–70, and >70 years), and gender quotas were set at a 50:50 male-to-female ratio. For geographical distribution, participants were categorised by settlement type and grouped as either urban (city/town) or rural (village/rural), in line with national population proportions. Participants were reimbursed for their time.

The aim was to achieve a total target sample size of at least 100 responses per health state description for robustness to allow for potential participant drop-off or incomplete data based on recommendations from the published literature and the EuroQol group [18,25].

### 2.4. Time-Trade-Off Study

The method used for estimating utilities for each of the health states was the time trade-off (TTO) approach, in line with the composite TTO protocol which was originally developed by the EuroQol Group for EQ-5D-5L utility tariff valuation [25]. The TTO tool used in this study was implemented via computer-assisted personal face-to-face interviews, and the incoming data were monitored throughout to ensure quality. The bespoke interview tool was built in Microsoft^®^ PowerPoint, based on EuroQol’s portable valuation technology (EQ-PVT) principles [26]. For each vignette, standard TTO utility elicitation methods were used, in which participants were asked to state whether they would prefer to live in the state of health described for 10 years, followed by death, or whether they would rather have a life in full health for a shorter duration (t). The value for t was iteratively varied between low and high values of time in best imaginable health until the point of indifference between the scenarios was found. TTO responses were converted to utility values on the scale of 0 to 1 by dividing the time stated in the best imaginable health (t) by 10.

The TTO interviews were held face to face by a trained interviewer/moderator using a laptop or tablet device between July 2022 and September 2022. Participants were asked to complete a consent form, followed by a set of demographic questions and utility-elicitation warm-up tasks (using the visual analogue scale [EQ VAS] thermometer to provide a 0–100 rating for each vignette) before conducting the formal TTO valuation exercise.

In the situations where participants indicated that they prefer immediate death rather than to live in the described health state for 10 years, the TTO exercise was adapted using the lead-time TTO approach, involving choices between a short life comprising t years in full health, and a longer life with 10 years in full health followed by 10 years in the disease state (for a combined duration of 20 years). Likewise, t was varied to find the point of indifference between the two choices. Utility values for these health states were calculated as U = (t − 10)/10, where the possible utility range is −1 to 1. Negative values arise when respondents are willing to give up part of the initial 10-year lead time to avoid living in the target health state, indicating that the health state is considered worse than death.

The main task entailed the valuation of all nine health state vignettes, in a fixed pre-specified order across all participants, which was designed to avoid presenting health states in ascending or descending severity order to reduce order bias. The order was intentionally mixed rather than presented in increasing severity to reduce potential anchoring to adjacent health states. The presentation order was HS3, HS1, HS6, HS4, HS5, HS2, HS-CG B, HS-CG A and HS-CG C. Each health state was valued using the TTO by >100 respondents across the sample.

Responses were excluded if (i) a participant gave the same health state valuation to 100% of health states or (ii) a participant chose the health state valuation of 1 (perfect health) for all health states (non-trading). This is in line with standard practice in TTO studies, and in line with the approach used to generate the EQ-5D-3L UK value set (which uses TTO-generated values) by the EuroQoL group [25,27].

### 2.5. Secondary Carer Sub-Study

In many rare diseases, more than one person is involved in providing informal care and/or incurs an HRQoL decrement as a result of providing unpaid care. This is particularly pertinent in EB, where assisting severe EB patients with dressing changes can take family members several hours a day and can have significant emotional impact, especially for parents of young children, due to the additional pain the patients often experience during wound care. To complement the primary carer utility values elicited from the main TTO study, a small exploratory sub-study was therefore designed to quantify the carer burden and estimate the utility impact in second carers. The sub-study consisted of an additional online questionnaire which was developed following the main TTO study, and underwent piloting with representatives from DEBRA UK, a UK patient advocacy group for EB. Following the finalisation of the survey materials, the questionnaire was disseminated via DEBRA UK to carers/family members of people living with EB and healthcare professionals experienced in EB treatment [28]. The questionnaire was self-completed online, using a survey link hosted by SurveyMonkey and made available in the members-only area of the DEBRA UK website during March–April 2023 [29]. This approach ensured that only verified members of DEBRA UK who had direct experience with EB could access and complete the survey, in order to maintain the integrity of the data collection process.

Only the most severe of the three carer perspective vignettes (HS6-CG3) corresponding to BSAP > 25% in the main TTO study was included in the secondary carer sub-study. This was based on feedback from DEBRA UK that this would be the health state that would be most likely to have more than one carer/family member involved in caring for a person with EB (e.g., due to very frequent bandage changes needed, and higher physical and emotional support required compared to relatively less severe states). Also, only one additional carer was considered (although there could be multiple additional people involved in caring and incurring a burden; for simplicity, the exercise was restricted to a second carer). The sub-study survey consisted of two core questions, where participants were first asked whether more than one informal carer would typically be involved for the specific carer vignette (Figure 2). Participants were then asked to consider circumstances in which a secondary carer was involved and asked to estimate the extent to which caregiving affected the HRQoL of the secondary carer relative to the primary carer. Responses were collected using a sliding scale where 0% indicates the secondary carer’s HRQoL/burden is not impacted at all, and 100% means their quality of life is impacted at least as much as the primary carer. The full questionnaire used is provided in Appendix B.

### 2.6. Statistical Analysis

For the main TTO study, as well as the secondary carer sub-study, descriptive statistics were used with mean utility and standard deviation (SD) reported. Frequencies and percentages were reported for categorical variables, such as demographic characteristics.

Paired *t*-tests were performed to test for differences in utilities elicited in the TTO study. Analysis of variance (ANOVA) and post hoc analyses with Bonferroni correction were conducted to compare utilities across age (‘18–39’ vs. ‘40+’ years), gender, and education level (‘with higher education’ vs. ‘without higher education’) subgroups.

A scenario analysis for the TTO study was also performed to assess the robustness of results. This primarily consisted of removing responses for participants who refused to trade life years during the TTO. The primary quality assurance mechanism was interviewer observation; moderators observed participant engagement and comprehension throughout the TTO task, noting any concerns regarding participant understanding or effort. Time-based thresholds were additionally applied as a supplementary check, including results for participants who responded in <1 min for all main TTO tasks and/or <3 min for all TTO examples, potentially indicating limits in the consideration given to the exercise. Participants identified through either of these checks were reviewed separately in a sensitivity analysis.

## 3. Results

### 3.1. Characteristics of the Main Study Participants

In the TTO full analysis sample of N = 120, including participants from the pilot study (*n* = 10; as vignettes were not altered following the pilot study, these data were deemed suitable for inclusion in the final analysis set), five non-traders were identified; these respondents either valued all vignettes as 1 (perfect health) or valued all vignettes as the same (i.e., they were non-trading). For all analyses and results, these non-traders were removed, resulting in a final analysis set of *n* = 115.

Table 1 describes the characteristics of the participants forming the final analysis set (*n* = 115). The majority were male (51.3%), and the mean (SD) age was 48.0 (17.0) years. Most respondents lived in an urban area (town 48.7%; city 38.3%) and were employed (full-time 42.6%; part-time 27.8%). On average, the highest level of education was sixth form/college (38.3%). Among the participants, 29.6% reported living with health problems; the most commonly reported health condition was fatigue (12.2%). The overall age distribution of the study sample was similar to the adult general population for the UK, albeit with a slightly lower proportion in the ‘60+ years’ category. The gender distribution of the enrolled TTO study sample was relatively closely matched to that of the general population of the UK; while the proportions differed by a few percentage points, this is not considered a substantial deviation. The sample characteristics appear broadly representative of the UK population, based on the 2021 census [24].

### 3.2. Patient and Primary Carer Utilities

The utility values estimated, based on the vignettes for each BSAP health state, are presented in Table 2. Generally, the mean patient utility decreased with increasing EB severity (greater BSAP), apart from between HS5 and HS6, where utility values were very similar. Carer utilities were higher than the equivalent patient health states, but also decreased as EB severity increased. Histograms representing the distribution of values for each patient and carer health state are presented in Appendix C.

*t*-tests confirmed that the utility differences between patient health states HS3, HS4 and HS5 were significant at the *p* < 0.01 level; however, at the lowest severity end (HS1 and 2) and at the highest severity end (HS5 and 6), the differences were not found to be significant, neither at *p* < 0.05 nor at *p* < 0.01 (Table 3). For the carer health state utility values (HS A, B and C), high levels of statistical significance were noted (at *p* < 0.01).

A scenario analysis was performed to assess the impact of excluding results from participants with rapid TTO completion (responded in <1 min for all main TTO tasks and/or <3 min for all TTO examples), resulting in a scenario analysis set of *n* = 93 participants following the removal of *n* = 22 rapid responders. Exclusion of these data had little impact on the overall utility estimates, with less than a 0.02 change in utility estimates observed across the health states (Table 4).

### 3.3. Secondary Carer Sub-Study

The exploratory sub-study to estimate the care-related disutility of a secondary informal carer, relative to that of a primary informal carer, for a patient in the most severe EB health state, consisted of *n* = 6 participants who completed the online survey. Respondents were either family members of a patient with EB (*n* = 3) or healthcare professionals with experience in the treatment of EB (*n* = 3).

**Figure 3:**
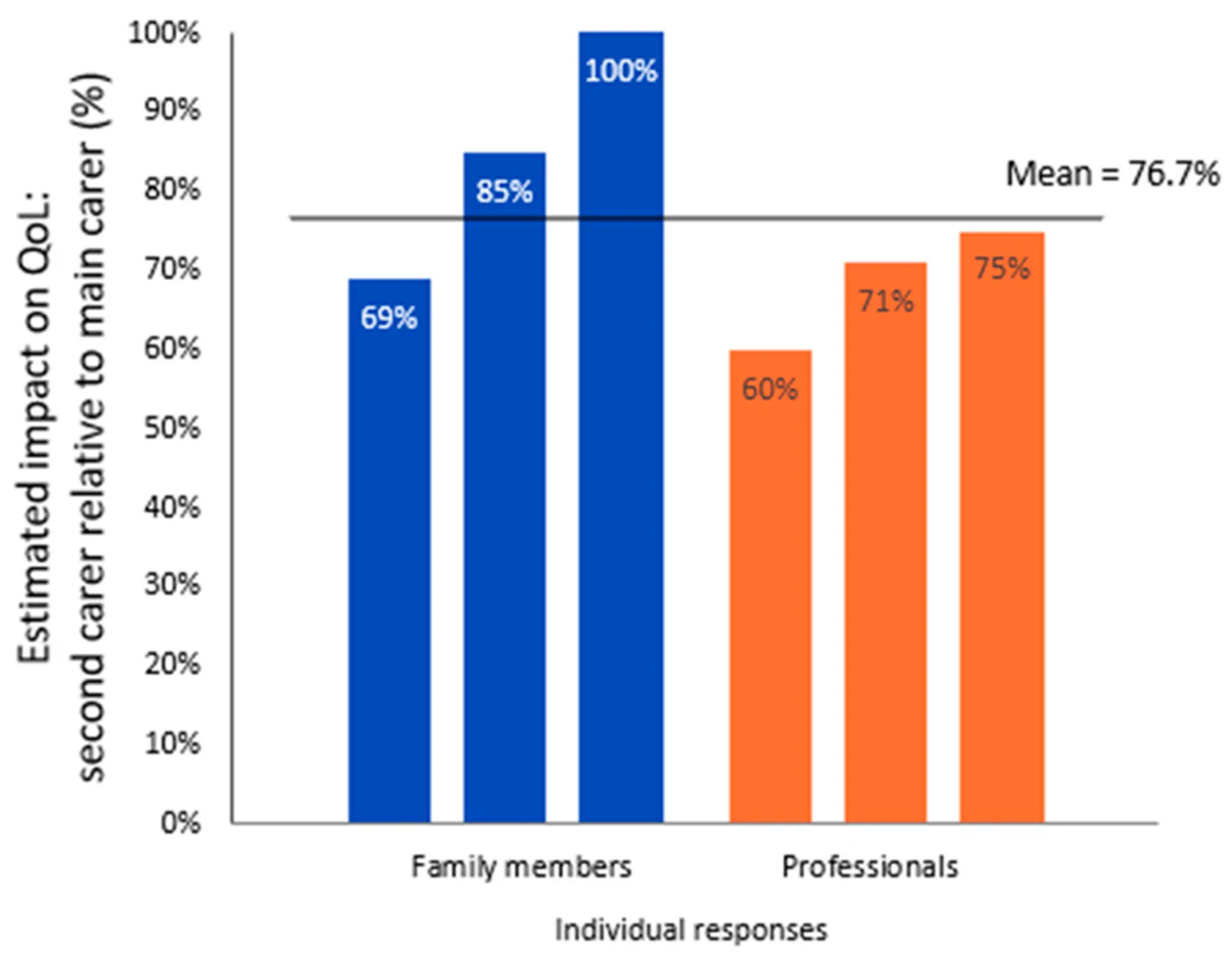
Relative HRQoL impact for an additional (second) carer: Individual responses by respondent type.

Individual responses by respondent type are shown in Figure 3. All estimates provided by healthcare professionals were below the mean value of 76.7%, with a lower average than that of the EB carer respondents, with one of the EB family member participants providing a 100% response, indicating that the secondary carer HRQoL was estimated to be impacted at the same level as the primary carer.

The age and gender characteristics of EB carers in the UK reported by Angelis et al. (2016), which identified a mean age of 41.5 years and a male-to-female ratio of 1:10, were used to derive an expected utility score of 0.91 for non-carers according to general population norms [30,31]. The disutility incurred by primary caregivers was defined as the absolute difference between the carer utility estimated in the TTO and the score expected in the general population, such that a disutility of 0.26 (0.91–0.64) was estimated for primary carers in HS-CG C.

**Figure 4:**
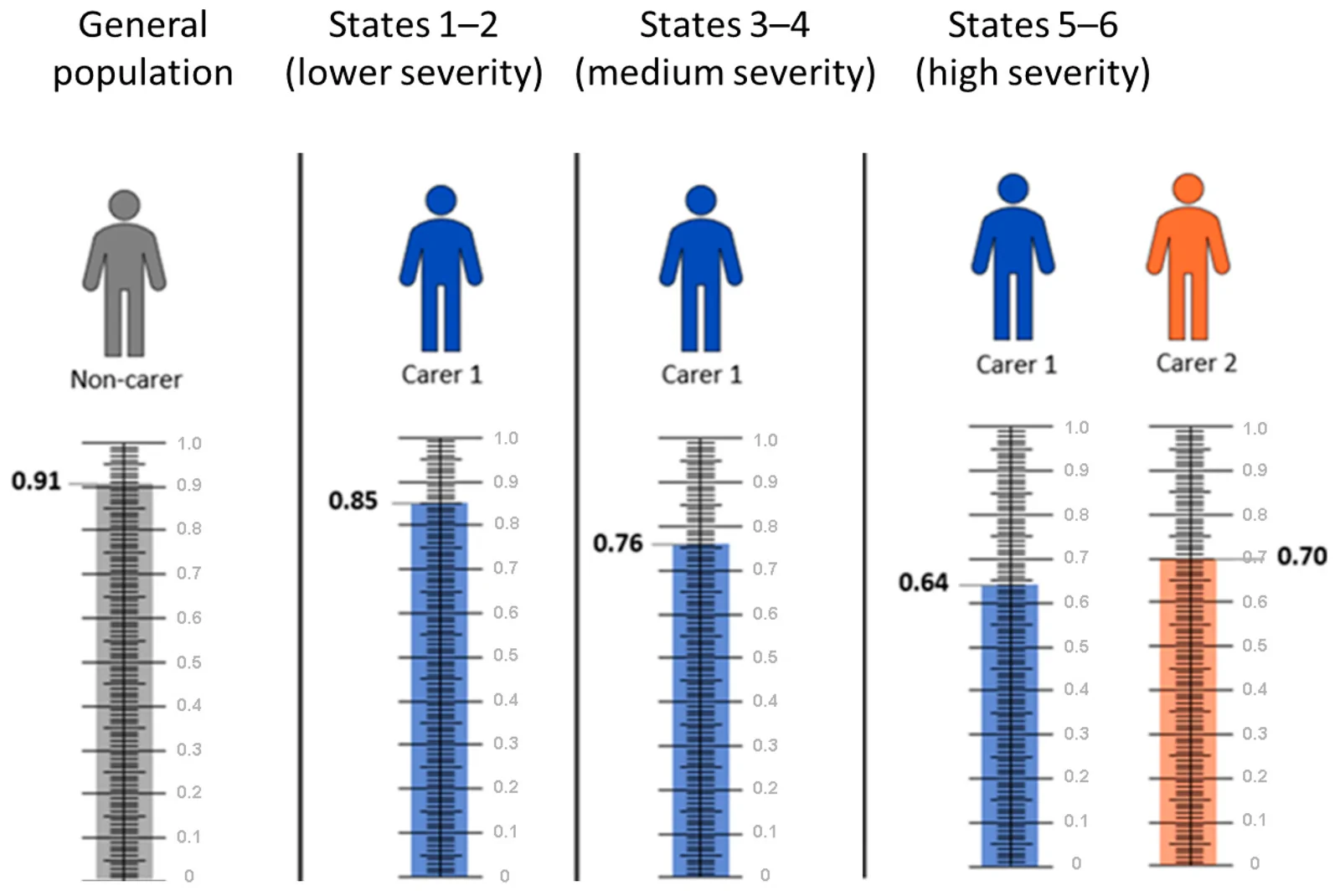
Estimated carer utility impact per health state.

Among second carers, disutility relative to the general population level was assumed to be 76.7% of the primary carer disutility based on results of the second carer sub-study. Applied to a primary carer disutility of 0.26 in HS-CG C, this resulted in an estimated second carer disutility of 0.20, corresponding to a second carer utility score of 0.70 compared to 0.64 for the primary carer, as shown in Figure 4 [30,31]. Second carer utilities for health states 1–4 were not examined in the exercise; hence, are not included in Figure 4.

## 4. Discussion

EB is a chronic disease that places a significant burden and quality of life impact on both patients and their informal carers, particularly in the most severe EB types. To our knowledge, this is among the first studies to quantify the HRQoL impact of living with EB through elicitation of utility values for patients as well as primary informal carers. The exploratory sub-study to assess relative burden in a secondary informal carer is also among the first, to our knowledge, in EB, or any rare disease, to quantify the relative burden and utility impact for a second unpaid carer in a family. Conducting the main TTO study in the UK general public means that the utility values generated are from a taxpayer/community perspective, in line with the principles of the National Health Service (NHS) whereby treatments are funded out of public tax revenues. The utility values were used in the economic evaluation of birch bark extract (Flisuvez^®^), the first licensed drug treatment for EB, to meet the evidence requirements of the NICE and Scottish Medicine Consortium (SMC) technology appraisal of this therapy [13]. Health state utility values that reflect the HRQoL of patients with different disease severities are essential for Quality Adjusted Life year (QALY) estimation needed in UK HTA, and our study has demonstrated a pragmatic approach to estimation of these utilities in a rare disease such as EB that is intended to meet NICE and SMC evidence requirements, and potentially as a basis for use in HTA in other cost-effectiveness markets that use QALYs as a core outcome metric.

The utility estimates generated for patients and primary carers demonstrate the significant potential HRQoL impact and burden associated with EB, with the estimated utilities decreasing as EB severity, proxied by BSAP states, increased. For the least severe BSAP state (up to 4%), the mean utility of 0.82 is below UK population norms, which have been estimated, using EQ-5D-3L TTO-derived values, to range from 0.94 for 18–24 year-olds to 0.85 for 45–54 year-olds [32]. Utility decreased with increasing BSAP severity up to HS5 (19–<25%), which was associated with an estimated utility of 0.53. The biggest utility decrease was between HS3 (BSAP: 8–10%) and HS4 (BSAP: 11–18%), with a statistically significant decrease from 0.76 to 0.61, respectively. Transition between these two BSAP health states is typically associated with a higher degree of malnutrition and anaemia, requirement for daily pain relief, moderate difficulty with eating and drinking, and sometimes requiring a wheelchair, as captured by the vignettes. In terms of the lowest severity end (HS1 and 2) and at the highest severity end (HS5 and 6), the differences were not found to be statistically significant but were for all other paired health state comparisons.

HS5 and HS6 are the most severe states with the highest body surface area covered by wounds; 19–<25% and ≥25%, respectively. These states are similar in terms of aspects such as being associated with a high risk of infection, a small risk of kidney issues, daily need for a high dose of painkillers, a frequent need for surgery for separating fused fingers or toes, regular screening for heart and kidney problems, and frequent negative emotions, as well as many aspects that also impact lower BSAP health states. Differences in HS6 (≥25% BSAP) compared to HS5 (19–<25% BSAP) include additional caregiving hours for dressing changes (4+ h vs. 2–4 daily), severe acute pain and itch vs. moderate pain and severe itch, and being unable to eat and drink or sleep normally vs. moderate difficulty with these activities. Despite this additional clinical burden, utility values for HS6 and HS5 were nearly identical (0.54 vs. 0.53), which may indicate, but does not confirm, a floor effect in perceived HRQoL at higher BSAP severity. While a floor effect is one possible explanation, whereby incremental differences in symptom severity become less distinguishable to participants when the overall health state is already substantially impaired, it is acknowledged that values of approximately 0.53 are not close to the lower bound of the utility scale (i.e., equivalent to death, or worse than death) and that TTO studies have successfully elicited distinct utility values for severe health states in other conditions. An alternative explanation is that the vignette descriptions for HS5 and HS6, whilst clinically distinct, may not have conveyed sufficiently differentiable HRQoL impacts to general public respondents unfamiliar with EB. It is also important to acknowledge that BSAP, whilst used here as the basis for health state definition due to its availability in the clinical data underpinning the aligned economic model, may not be the most clinically intuitive or comprehensive descriptor of EB severity. Although the vignettes themselves describe a broad range of symptoms and impacts beyond wound coverage alone, the underlying health state boundaries are defined by BSAP thresholds, which may not fully capture the complexity of disease burden at the severe end of the spectrum, potentially limiting the perceived differentiation between adjacent severe health states. These findings are acknowledged as limitations of the study, and future research using a larger sample, alternative instruments for defining health state severity, or revised vignette descriptors may better capture utility differences at the severe end of the severity spectrum.

Estimated carer utilities were higher than the equivalent patient health states, as is often reported in carer utility literature, with values of 0.85, 0.76, and 0.64 for being the carer of a DEB/JEB patient with 5–7%, 11–18% and >25% wound BSAP, respectively. However, all the carer utility values fell below UK general population norms [32], demonstrating the detrimental impact associated with the burden of being the primary carer for a person living with EB. The differences in paired health state comparisons were all statistically significant, supporting the high impact as EB severity increases.

Although there are no prior TTO-based vignette studies in EB, other studies have explored the HRQoL impact and burden associated with EB through different methods [4,5,12,22,33,34]. The 2023 EB Insights study, commissioned by the patient body DEBRA UK, demonstrates the substantial morbidity associated with living with EB and the impact on patient HRQoL, reducing the average utility (measured by the EQ-5D) on a “typical day” to 0.601 for DEB and 0.483 for JEB [35]. The study also highlights that on the patient’s “worst day”, the average utility for DEB patients is 0.141 and 0.092 for JEB patients, highlighting the significant burden and HRQoL impacts of the disease. The utility values estimated for DEB and JEB patients on a “typical day” from the EB Insights study (weighted average: 0.542) are numerically similar to, though not directly comparable with, estimates from our TTO study given differences in elicitation method and disease-state definition. The average utility value in our TTO study was 0.675, compared to the EB Insights weighted average of 0.542. While the distribution of patients across BSAP health states in the EB Insights study is unknown, the lower utility values may reflect a more severe patient population or real-world disease burden compared to the standardised vignettes used in our TTO methodology. Quantitative informal carer HRQoL data were not collected in the EB Insights study; however, qualitative data demonstrated that carers reported a moderate to high impact on various aspects of life. These findings align with carer utility values reported by Angelis et al. (2022), where the average utility across 46 adult EB carers in the UK was 0.713 [36]. This value is broadly consistent with the carer utility values elicited in our TTO study: 0.64 for primary carers and 0.70 for additional carers. While the consistency between Angelis et al. and the TTO exercise described in this manuscript provides support for the face validity of the carer utility estimate, it is acknowledged that this does not constitute formal convergent validity, given the differences in methods, populations, and health state definitions between the two studies.

A potential limitation of the TTO is that order effects may have been introduced based on vignettes being presented to participants in a fixed order, rather than being randomised. Floor effects may have constrained the lower range of possible values, particularly for the most severe health states (HS5 and HS6), restricting participants from expressing more severe disutilities. The vignettes were also developed based on severity associated with EB sub-types of DEB and JEB, which are severe sub-types of EB. This was due to the objective of using the values for HTA purposes in the economic evaluation and NICE appraisal of birch bark extract [13], which is indicated in these sub-types. Hence, the values elicited do not directly apply to other EB sub-types, including EBS and KEB.

Estimation of the utility impact of disease on additional informal carers than the primary carer in a family (e.g., partner or sibling) is a hotly debated topic in HTA in the UK, and one where there is little robust evidence of that impact. Carer disutility has been an important component of economic evaluations performed in rare diseases for HTA, with 15 of 19 NICE Highly Specialised Technology (HST) appraisals performed as of June 2022 including this element, although primarily with a focus on the impact on one informal carer [37]. Eight of the HST submissions reviewed by Noble-Longster et al. (2022) included the utility impact of more than one carer, and five of these considered a different number of carers across different health states [37]. However, none of the estimates included in these HSTs has been evidence/data based. Our small sub-study has attempted to collect data and quantify the burden and associated relative utility burden for second carers in EB. It was added to the main primary carer TTO study based on feedback provided by the NICE Evidence Assessment Group on the utility values used in the economic model during the NICE technology appraisal for birch bark extract (HST28) for treating partial thickness wounds in patients with DEB and JEB [13].

The estimated relative burden of a second carer of a person with severe DEB/JEB (≥25% BSAP) was 76% of that of the primary carer, and the estimated utility of 0.70 compared to 0.64 for the primary carer indicates the significant impact that caring for someone with severe EB has on additional carers. In the sub-study, there was a disparity between the estimates elicited by the three EB secondary carers compared to those from the three EB healthcare professionals as proxy respondents. The EB secondary carers reported a higher relative burden than the healthcare professionals, with an average of 84.7%, compared to 68.7% for the three healthcare professionals. Whilst healthcare professionals bring valuable clinical expertise to EB care, they are unlikely to have full insight into the constant, round-the-clock nature of care provision and burden within the family unit, which likely explains healthcare professional participants valuing the secondary carer burden less severely than the secondary carers themselves. The average estimated burden impact from this study may plausibly be an underestimate of the value had only EB family members completed the exercise, though this remains speculative given the very small number of respondents in each group (*n* = 3).

Although an initial exploratory study with a small sample was conducted for one health state due to time limitations, the second carer sub-study represents a primary attempt to collect data and quantify this burden and to estimate utilities, providing an important first step in exploring the relative HRQoL impact of EB and rare diseases generally where informal care is received from more than one family member. The approach of modelling a secondary carer experiencing 77% of the QALY loss of the primary carer was accepted by the EAG and NICE as valid evidence for inclusion in the Birch Bark extract economic model and appraisal (HST28), and so could inform future economic evaluations of novel therapies for EB, as well as for other rare diseases where more than one family member provides unpaid care. However, in terms of precision of the estimates, it is limited by the small sample size and health state coverage, and so to increase robustness requires additional data collection in EB family members to validate the relative burden estimates across the most severe but also less severe EB health states not covered in our sub-study, for one or more additional informal carers. Further research to better understand the relationship between primary and secondary carer utilities, and how these might vary with factors such as patient age, disease severity (i.e., total BSAP coverage), and available support systems, could provide valuable insights for future health economic evaluations in EB.

## 5. Conclusions

This study has demonstrated the impact of increasing EB severity on both patient and informal carer HRQoL. HRQoL has been assessed in the form of utility values that can be used as inputs to economic evaluations of new EB therapies for HTA purposes in the UK and other cost-effectiveness-based drug reimbursement markets. The values reported here have previously been used in UK technology appraisals of the first licensed and reimbursed therapy for EB, Birch Bark extract [13,15]. The addition of an exploratory sub-study to explore the relative impact of severe EB on secondary carers provided further insights and represents the first attempt to quantify this impact in terms of relative burden and utility for use in health economic analyses. However, as the sub-study involved a small sample, further research with a larger sample is needed to enhance the robustness of the estimates.

## Figures and Tables

**Table 1:** Characteristics of the study participants (N = 115).

Characteristic	N (%)
*Gender, n (%)*	
Male	59 (51.3%)
Female	55 (47.8%)
Prefer not to say	1 (0.9%)
*Age, mean (SD)*	
	48.0 (17.0)
*Age categories (years), n (%)*	
18–25	14 (12.2%)
26–40	32 (27.8%)
41–55	29 (25.2%)
56–70	27 (23.5%)
>70	13 (11.3%)
*Living area, n (%)*	
Rural	3 (2.6%)
Village	12 (10.4%)
Town	56 (48.7%)
City	44 (38.3%)
*Highest education level, n (%)*	
Secondary school	33 (28.7%)
Sixth Form/College	44 (38.3%)
Bachelor’s degree	33 (28.7%)
Master’s/Doctoral degree	4 (3.5%)
Other	1 (0.9%)
*Employment status, n (%)*	
Full-time employed	49 (42.6%)
Part-time employed	32 (27.8%)
Retired	23 (20.0%)
Student	8 (6.9%)
Unemployed	2 (1.7%)
Full-time carer	1 (0.9%)
*Health problems reported, n (%)*	
Yes	34 (29.6%)
*Detailed health problems reported, n (%)*	
Fatigue	14 (12.2%)
Pain	11 (9.6%)
High blood pressure	11 (9.6%)
Diabetes	11 (9.6%)
Breathing problems	9 (7.8%)
Anxiety	9 (7.8%)
Osteoarthritis	7 (6.1%)
Insomnia	6 (5.2%)
Depression	4 (3.5%)

**Table 2:** Modelled utility values for patient and primary carer health states.

Patient Health States, (BSAP %)	Modelled Patient Utility Values Mean (SD) (N = 115)	Carer Health State, (BSAP %)	Modelled Carer Utility Values Mean (SD) (N = 115)
HS1 (up to <5%)	0.82 (0.2)	HS2-CG A (5–<8%)	0.85 (0.2)
HS2 (5–<8%)	0.79 (0.2)		
HS3 (8–<11%)	0.76 (0.2)	HS4-CG B (11–<19%)	0.76 (0.2)
HS4 (11–<19%)	0.61 (0.3)		
HS5 (19–<25%)	0.53 (0.3)	HS6-CG C (≥25%)	0.64 (0.3)
HS6 (≥25%)	0.54 (0.3)		

***Abbreviations:*** *BSAP, Body surface area percentage; HS1, Health State 1 (patient perspective)-BSAP up to 4%; HS2, Health State 2 (patient perspective)-BSAP 5–7%; HS3, Health State 3 (patient perspective)-BSAP 8–10% (HS3); HS4, Health State 4 (patient perspective)-BSAP 11–18%; HS5, Health State 5 (patient perspective)-BSAP 19–24%; HS6, Health State 6 (patient perspective)-BSAP ≥25%; HS-CG A, Health State A (caregiver perspective)-BSAP 5–7%; HS2-CG B, Health State B (caregiver perspective)-BSAP 11–18%; HS-CG C, Health State C (caregiver perspective)-BSAP ≥25%; SD, standard deviation.*

**Table 3:** Full analysis set paired comparisons (N = 115).

Health States (BSAP %)	Average Decrement	*t*-Test Two-Tailed *p*-Value
Patient perspective:		
HS1 vs. HS2	0.03	0.06
HS2 vs. HS3	0.03	0.125
HS3 vs. HS4	0.15	0.0001 **
HS4 vs. HS5	0.08	0.0001 **
HS5 vs. HS6	−0.01	0.382
Carer perspective:		
HS-CG A vs. HS-CG B	0.09	0.0001 **
HS-CG B vs. HS-CG C	0.12	0.0001 **

***Abbreviations:*** HS1, Health State 1 (patient perspective)-BSAP up to 4%; HS2, Health State 2 (patient perspective)-BSAP 5–7%; HS3, Health State 3 (patient perspective)-BSAP 8–10% (HS3); HS4, Health State 4 (patient perspective)-BSAP 11–18%; HS5, Health State 5 (patient perspective)-BSAP 19–24%; HS6, Health State 6 (patient perspective)-BSAP ≥25%; HS-CG A, Health State A (caregiver perspective)-BSAP 5–7%; HS2-CG B, Health State B (caregiver perspective)-BSAP 11–18%; HS-CG C, Health State C (caregiver perspective)-BSAP ≥25%. **Note:** Significant differences at the p < 0.01 are marked with **.

**Table 4:** Scenario analysis with rapid responders removed.

Health States (BSAP %)	Full Analysis Set (N = 115) Utility Values Mean (SD)	Scenario Analysis (*n* = 93) Utility Values Mean (SD)
Patient perspective:		
HS1 (up to 4%)	0.82 (0.2)	0.82 (0.2)
HS2 (5–7%)	0.79 (0.2)	0.81 (0.2)
HS3 (8–10%)	0.76 (0.2)	0.76 (0.2)
HS4 (11–18%)	0.61 (0.3)	0.60 (0.3)
HS5 (19–24%)	0.53 (0.3)	0.52 (0.3)
HS6 (≥25%)	0.54 (0.3)	0.53 (0.3)
Caregiver perspective:		
HS-CG A (5–7%)	0.85 (0.2)	0.85 (0.2)
HS-CG B (11–18%)	0.76 (0.2)	0.76 (0.2)
HS-CG C (≥25%)	0.64 (0.3)	0.63 (0.3)

***Abbreviations:*** *BSAP, Body surface area percentage; HS1, Health State 1 (patient perspective)-BSAP up to 4%; HS2, Health State 2 (patient perspective)-BSAP 5–7%; HS3, Health State 3 (patient perspective)-BSAP 8–10% (HS3); HS4, Health State 4 (patient perspective)-BSAP 11–18%; HS5, Health State 5 (patient perspective)-BSAP 19–24%; HS6, Health State 6 (patient perspective)-BSAP ≥25%; HS-CG A, Health State A (caregiver perspective)-BSAP 5–7%; HS2-CG B, Health State B (caregiver perspective)-BSAP 11–18%; HS-CG C, Health State C (caregiver perspective)-BSAP ≥25%; SD, standard deviation.*

**Figure A1:**
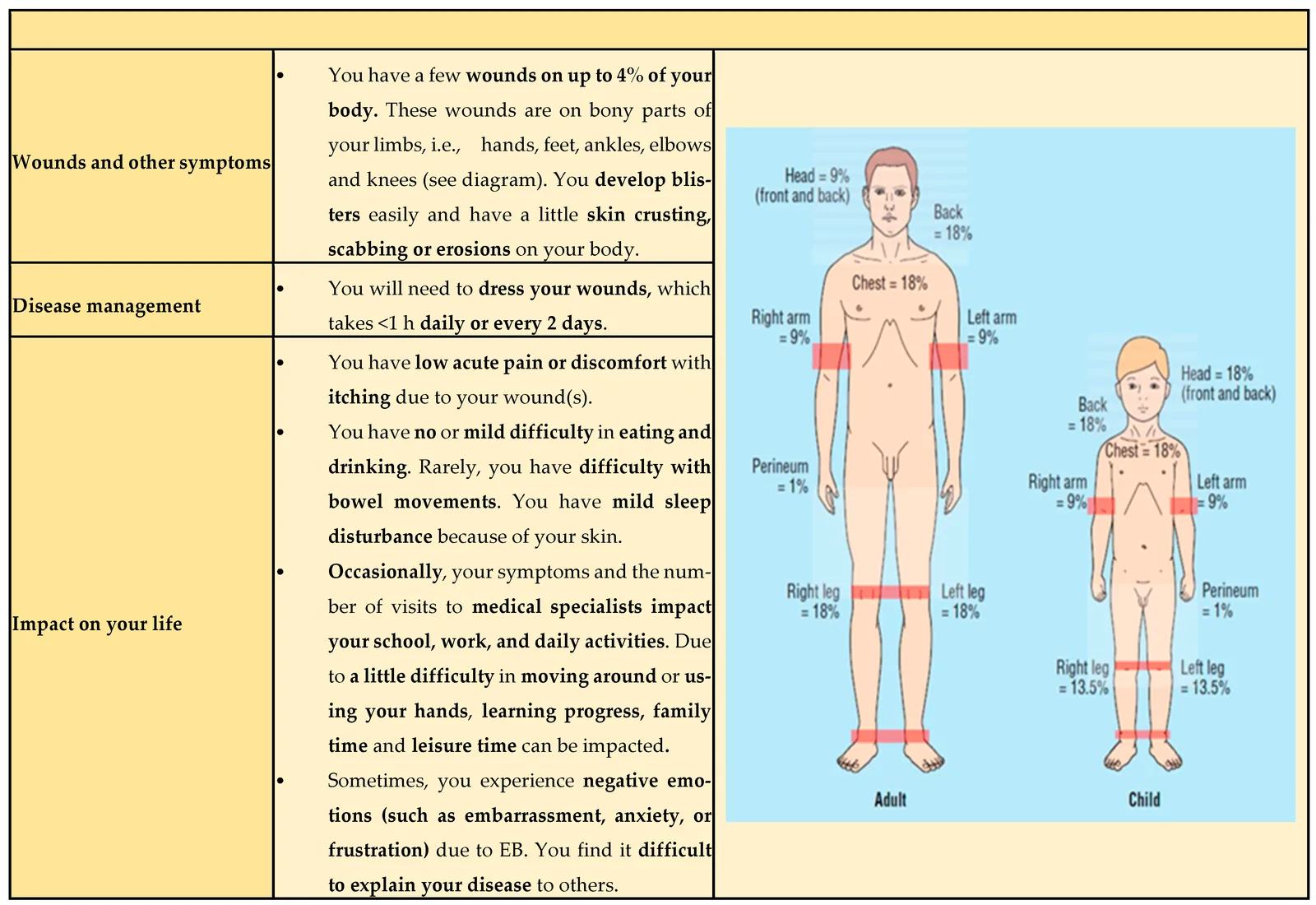
Health State 1 (patient perspective)—with wounds covering up to 4% of the body (HS1).

**Figure A2:**
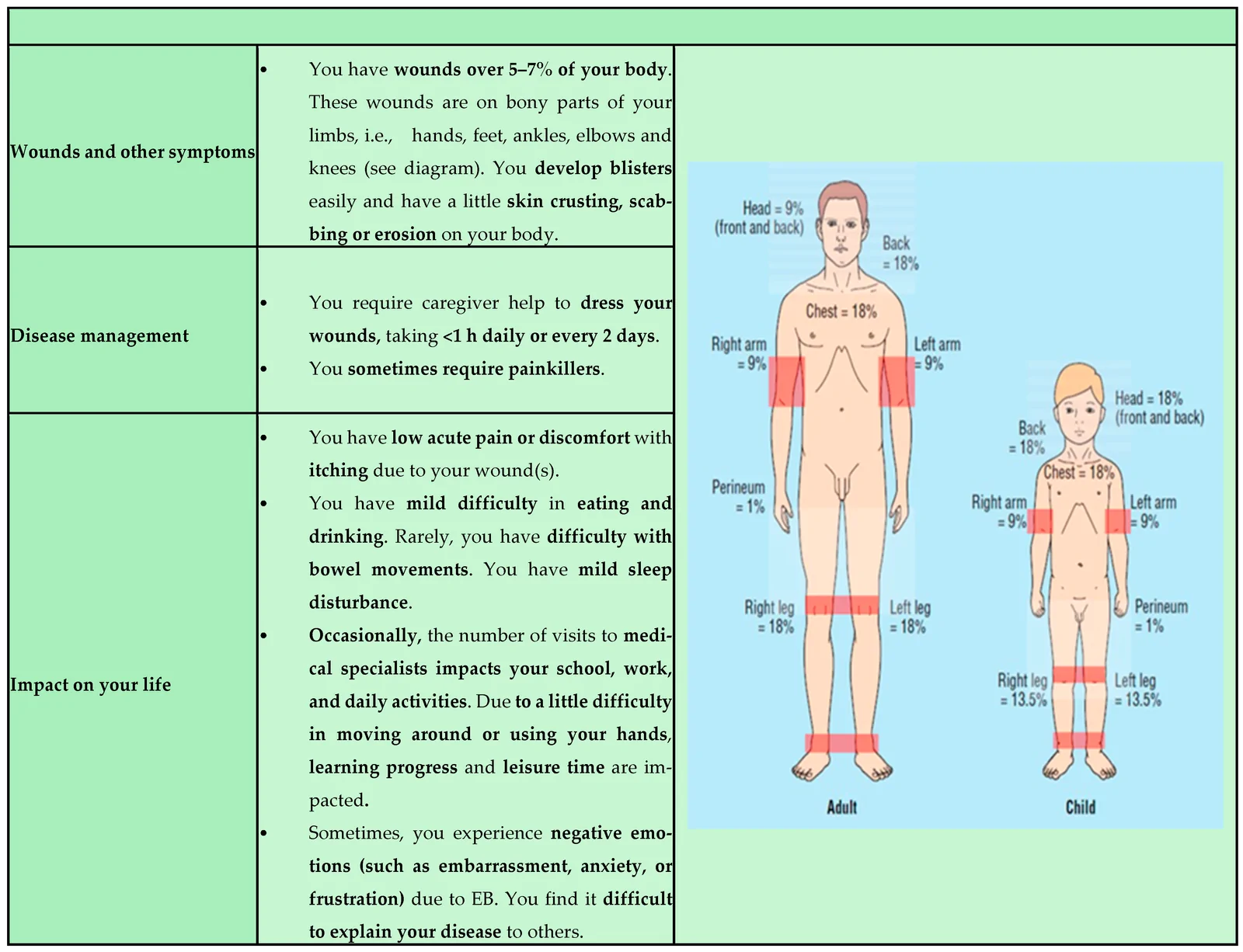
Health State 2 (patient perspective)—with wounds covering 5–7% of the body (HS2).

**Figure A3:**
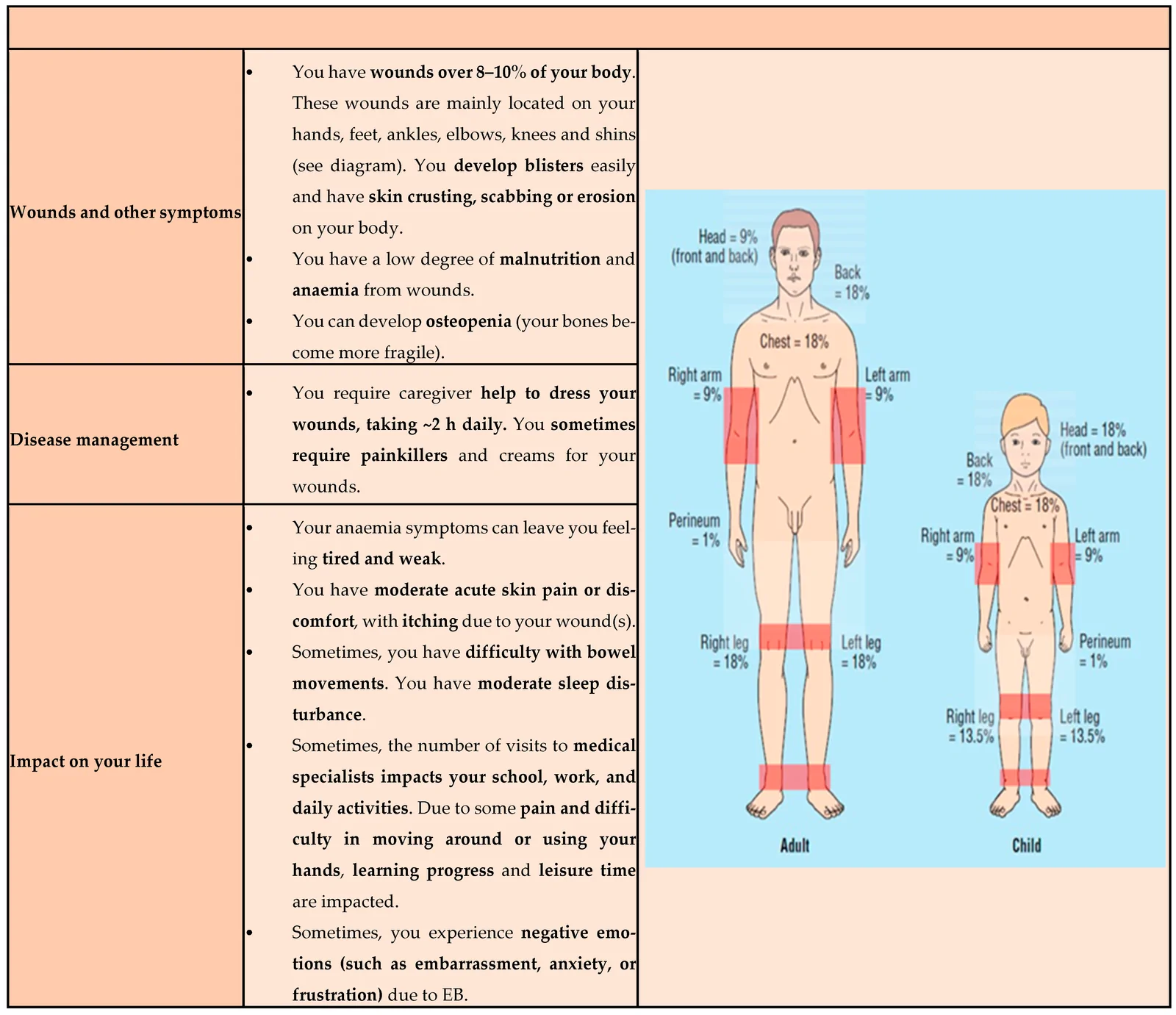
Health State 3 (patient perspective)—with wounds covering 8–10% of the body (HS3).

**Figure A4:**
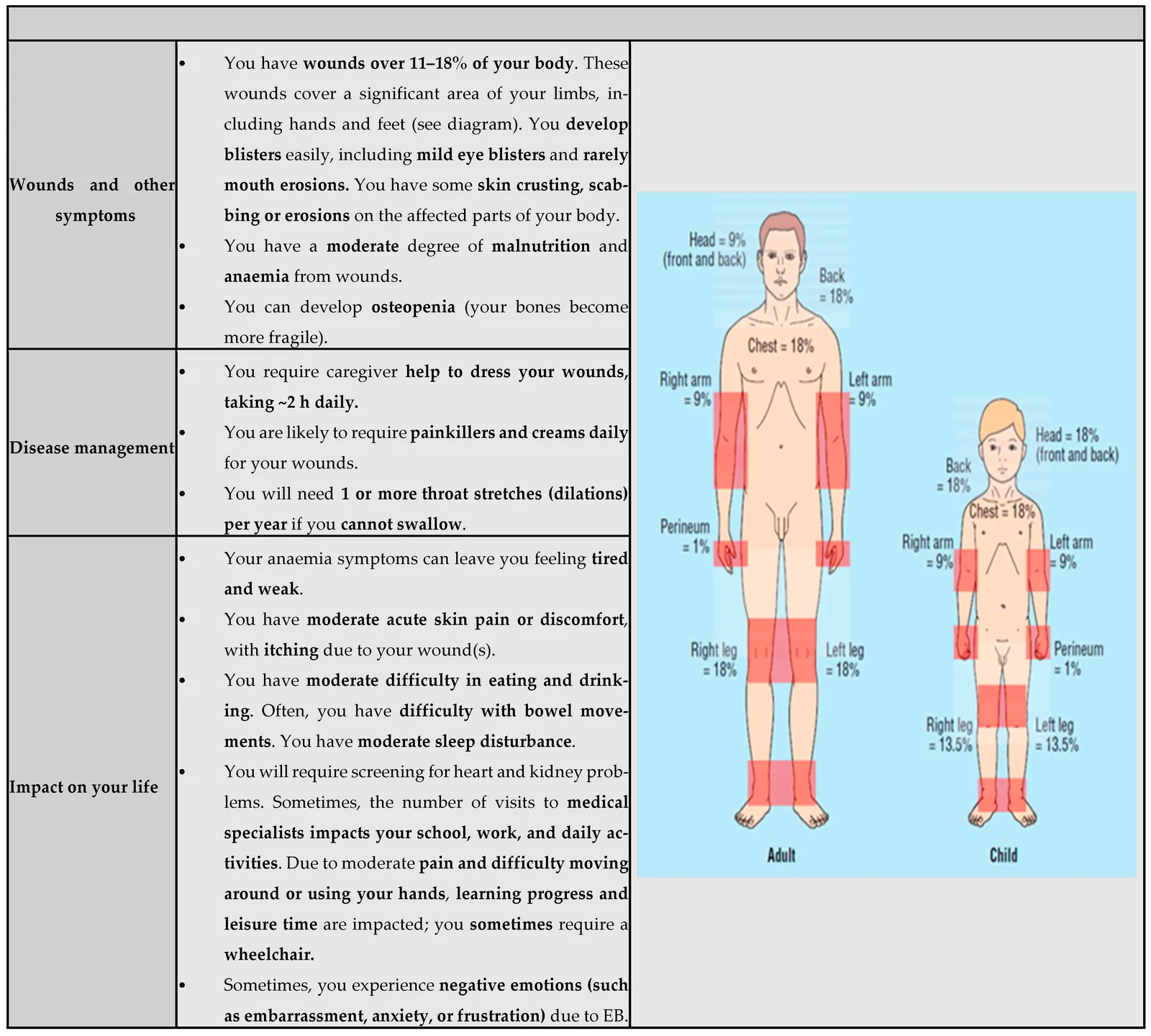
Health State 4 (patient perspective)—with wounds covering 11–18% of the body (HS4).

**Figure A5:**
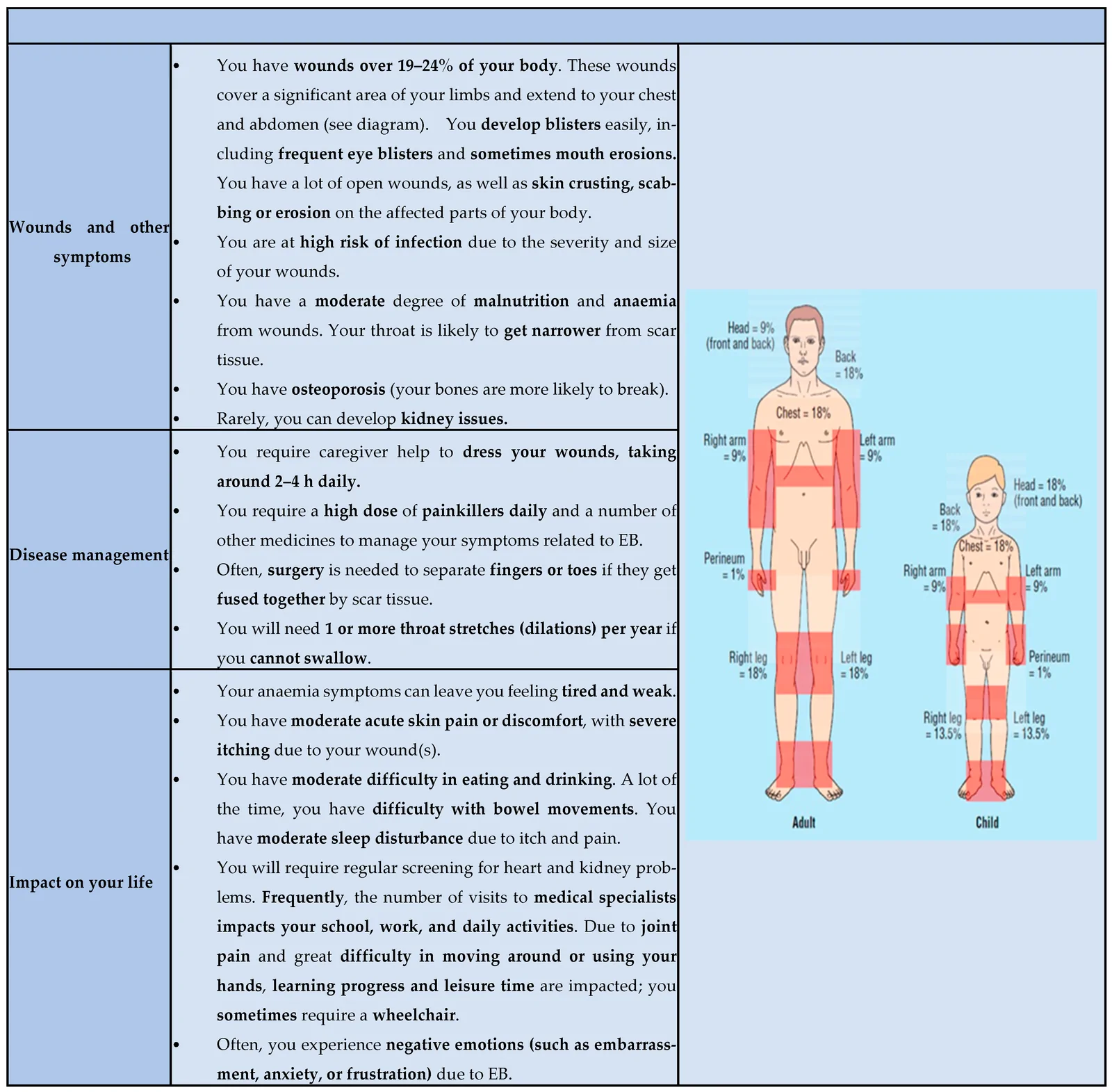
Health State 5 (patient perspective)—with wounds covering 19–24% of the body (HS5).

**Figure A6:**
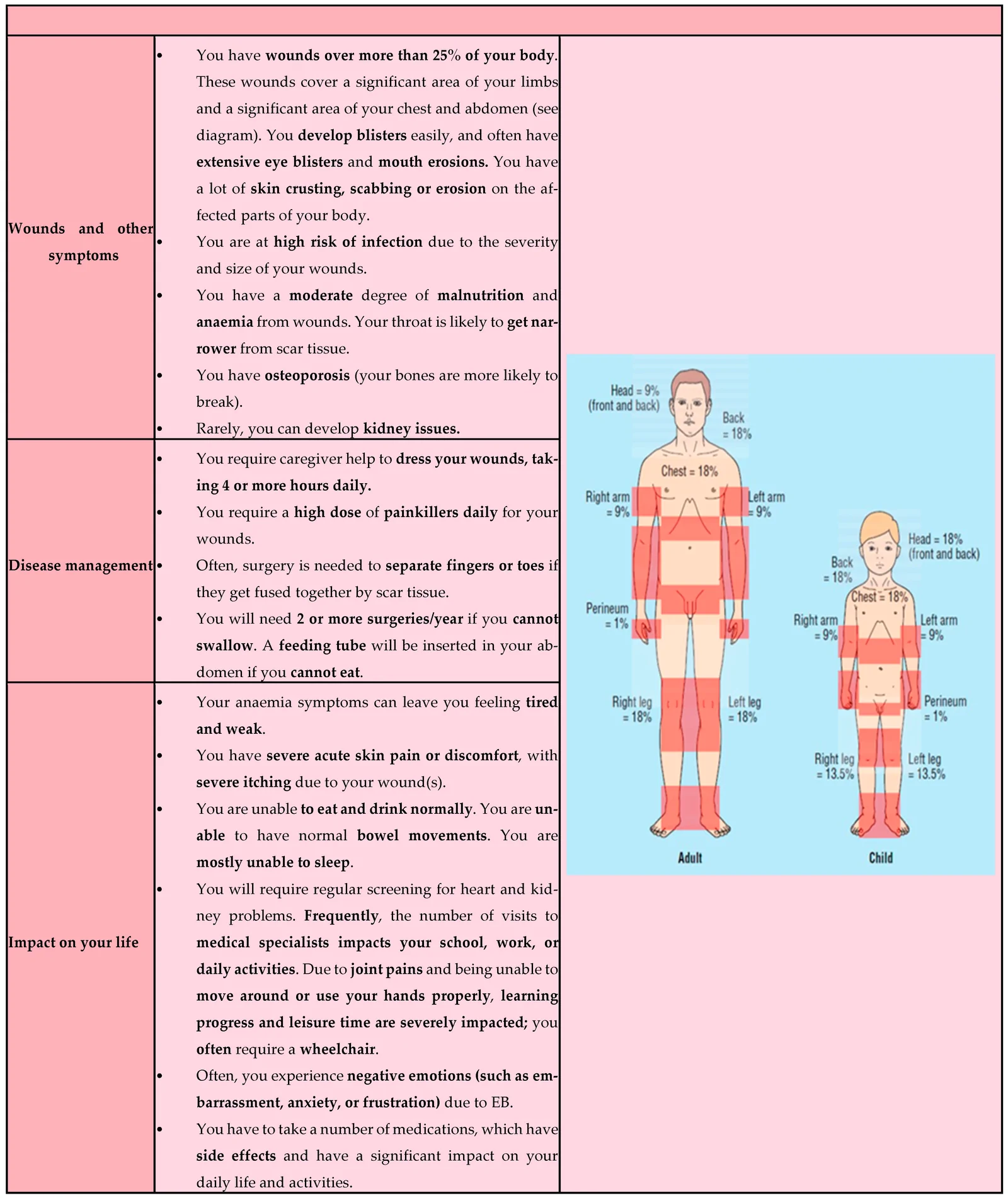
Health State 6 (patient perspective)—with wounds covering ≥25% of the body (HS6).

**Figure A7:**
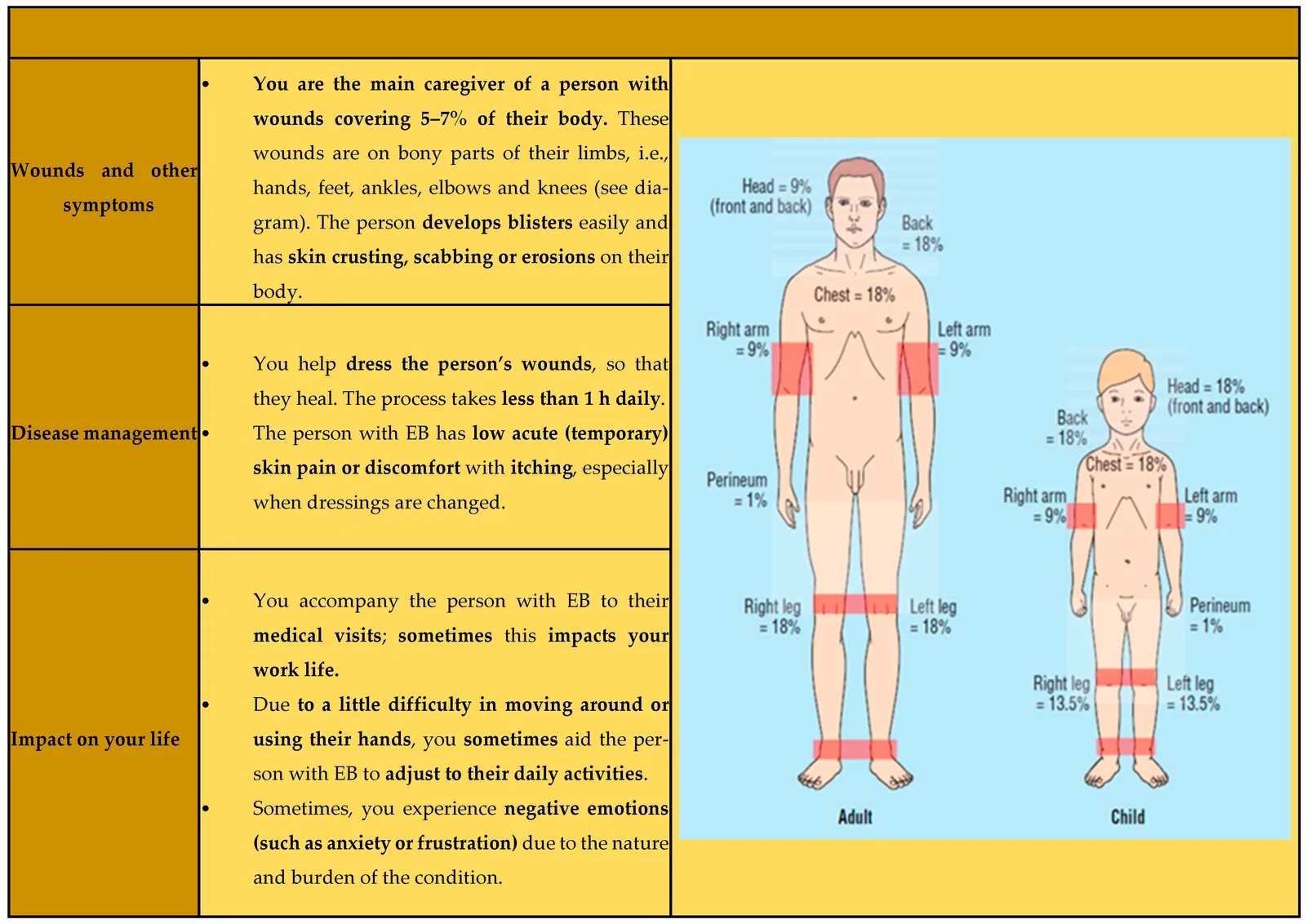
Health State A (caregiver perspective)—with wounds covering 5–7% of the patient’s body (HS-CG A).

**Figure A8:**
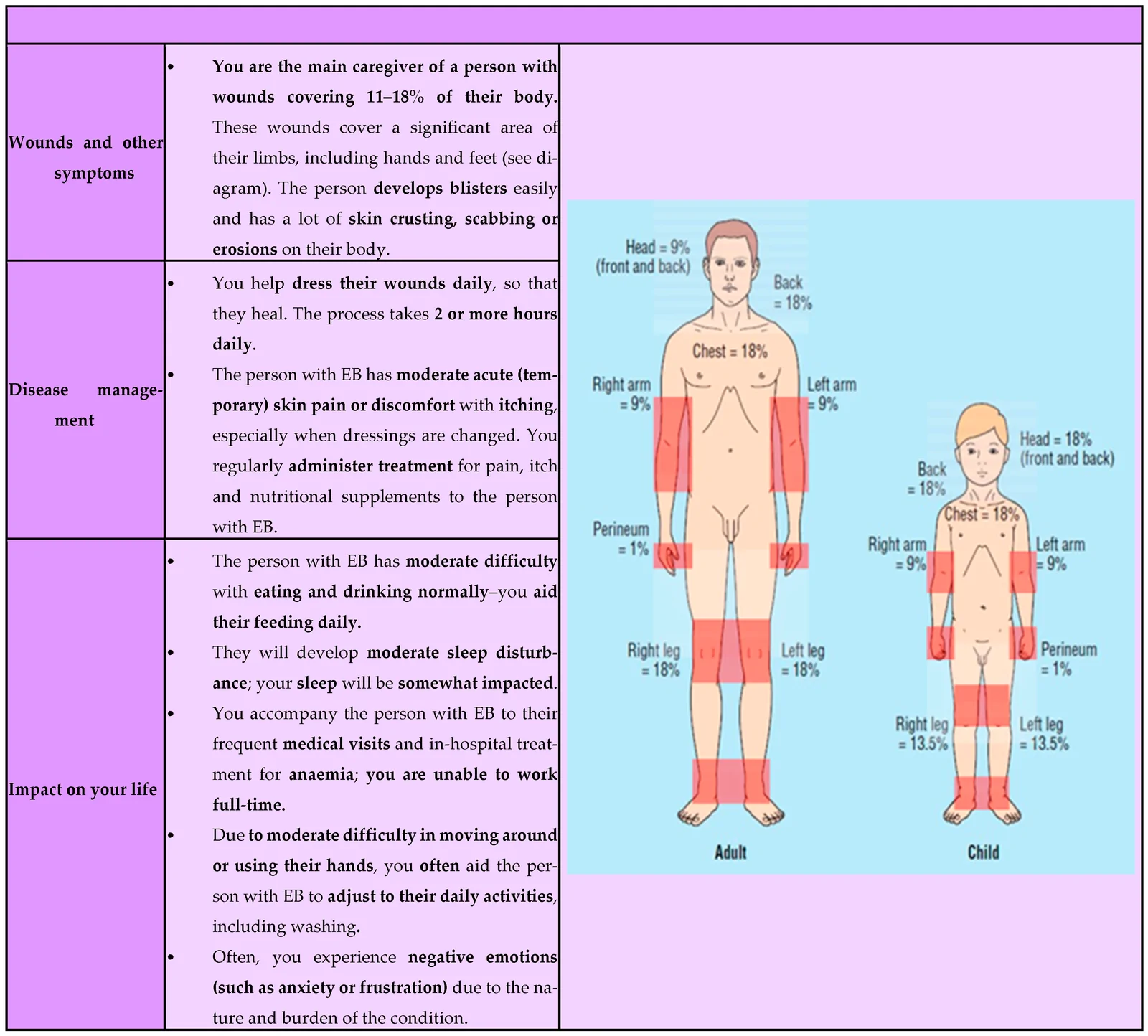
Health State B (caregiver perspective)—with wounds covering 11–18% of the patient’s body (HS2-CG B).

**Figure A9:**
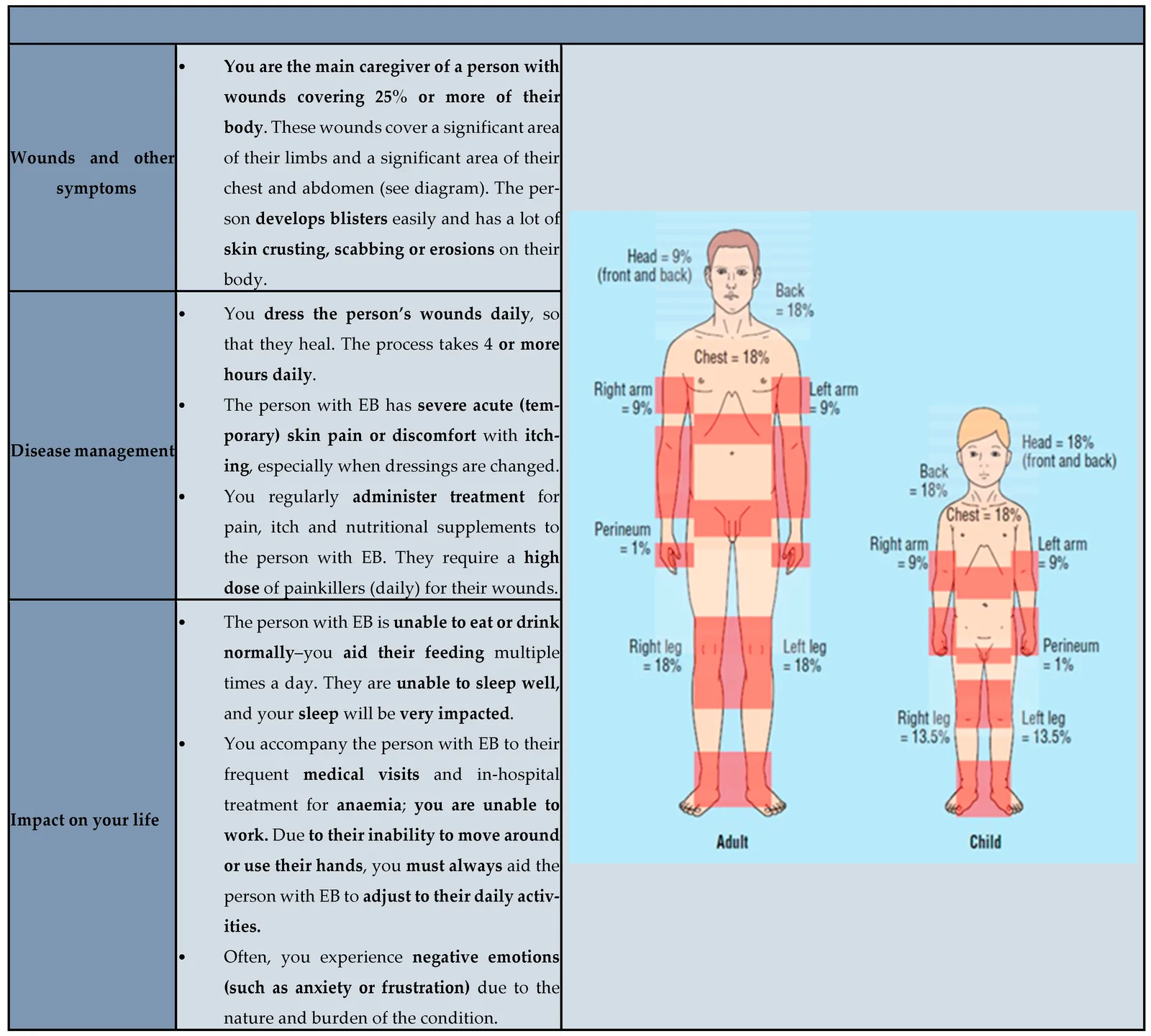
Health State C (caregiver perspective)—with wounds covering ≥25% of the patient’s body (HS-CG C).

**Figure A10:**
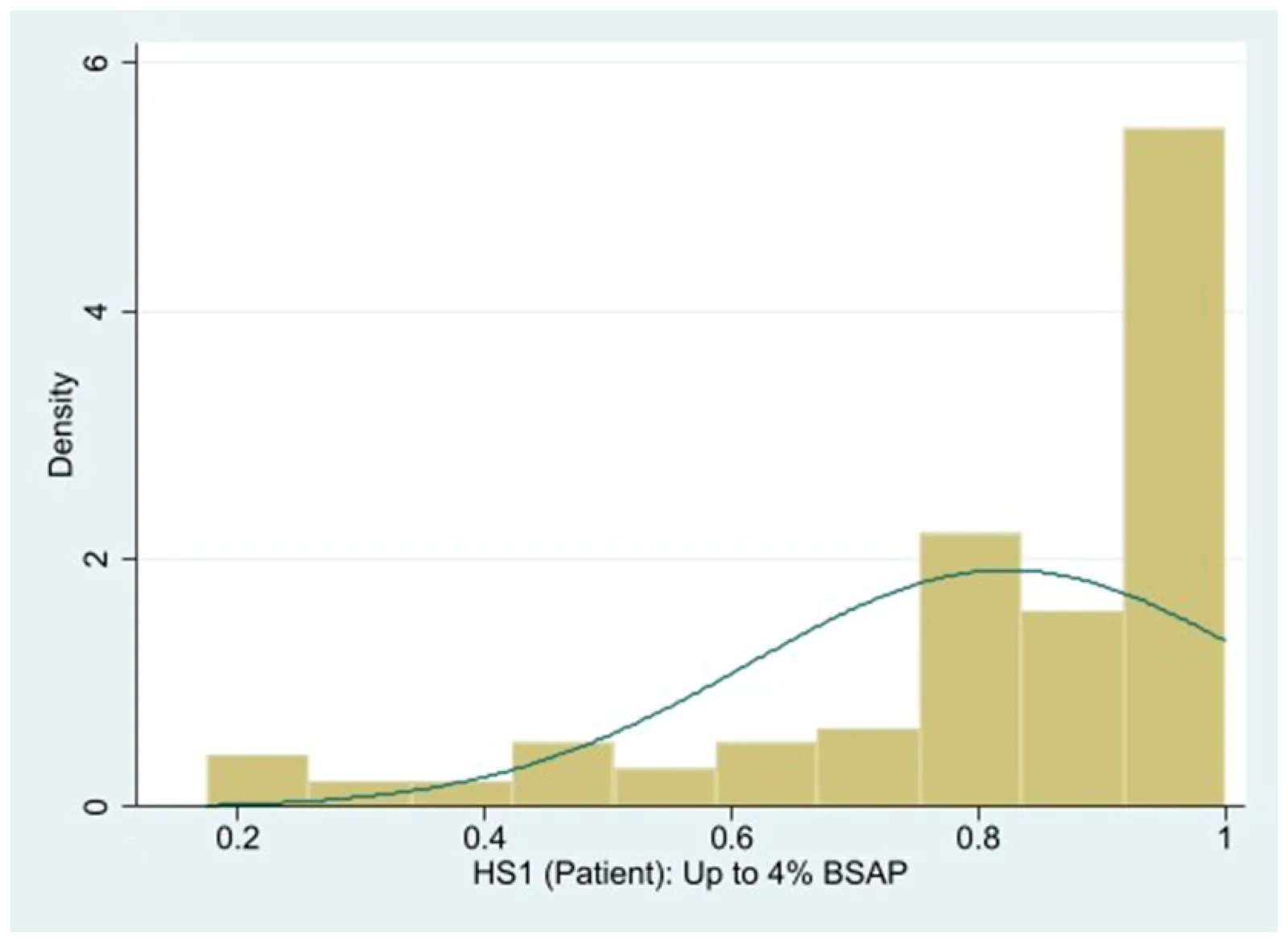
Health State 1.

**Figure A11:**
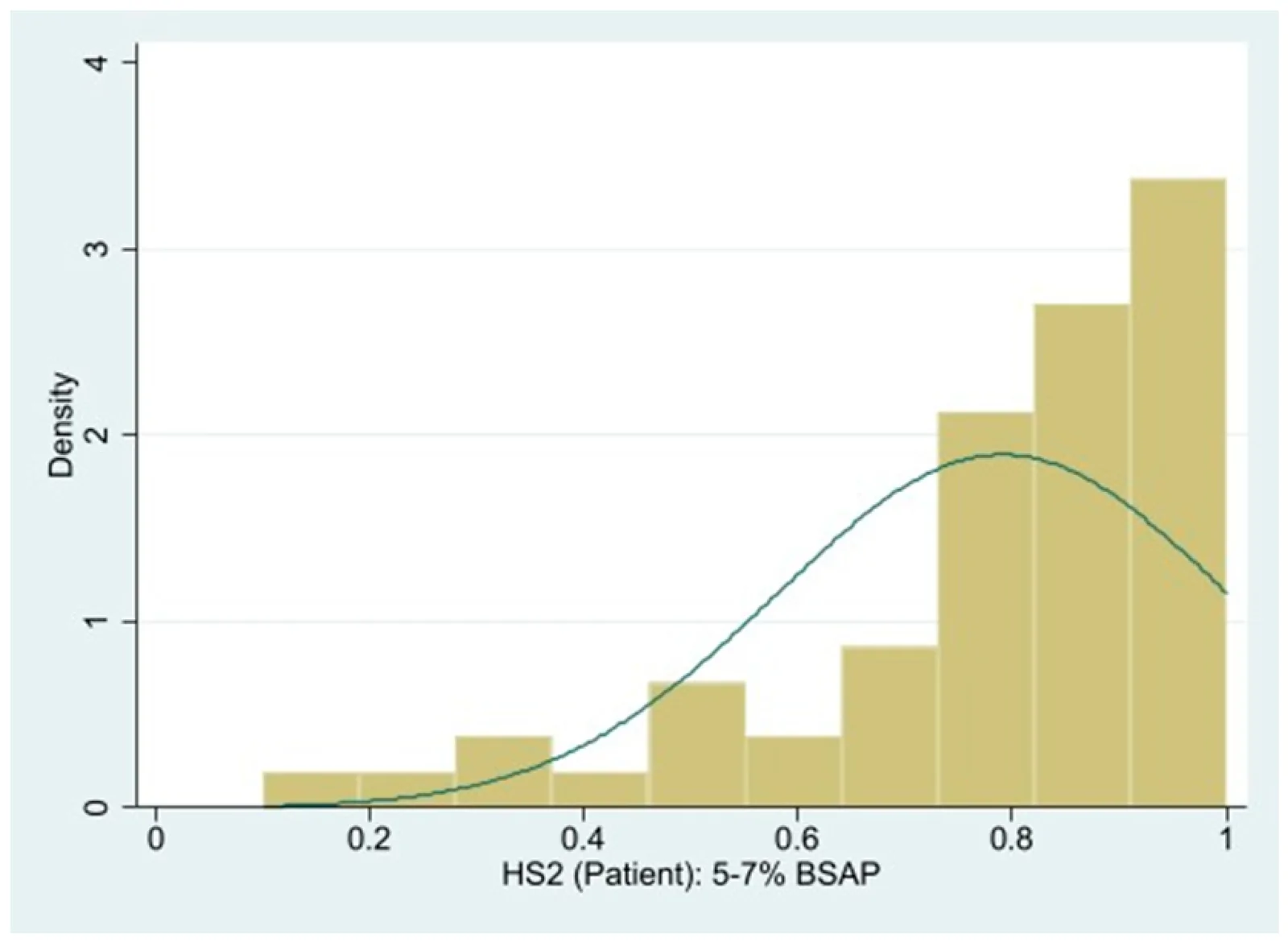
Health State 2.

**Figure A12:**
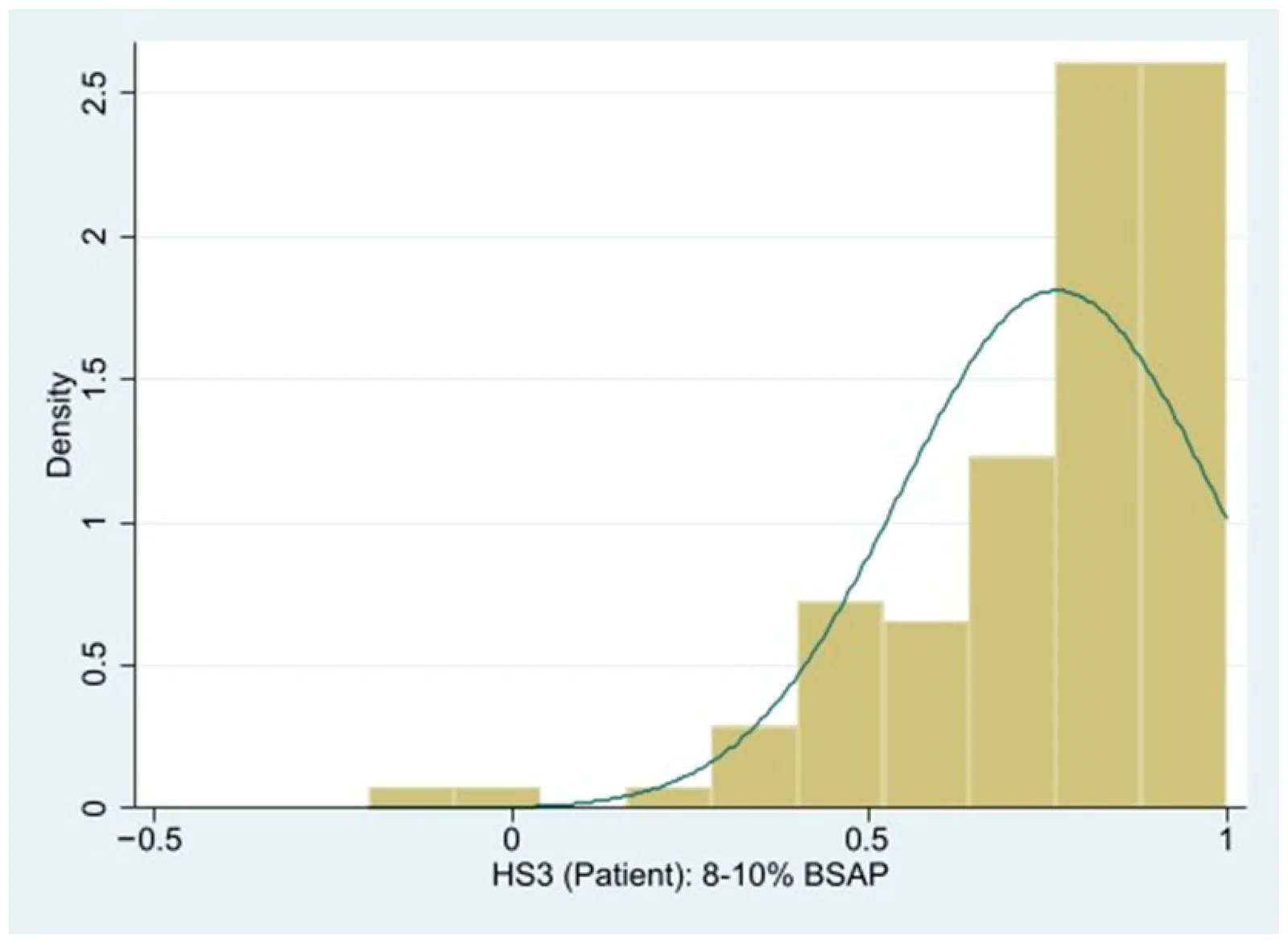
Health State 3.

**Figure A13:**
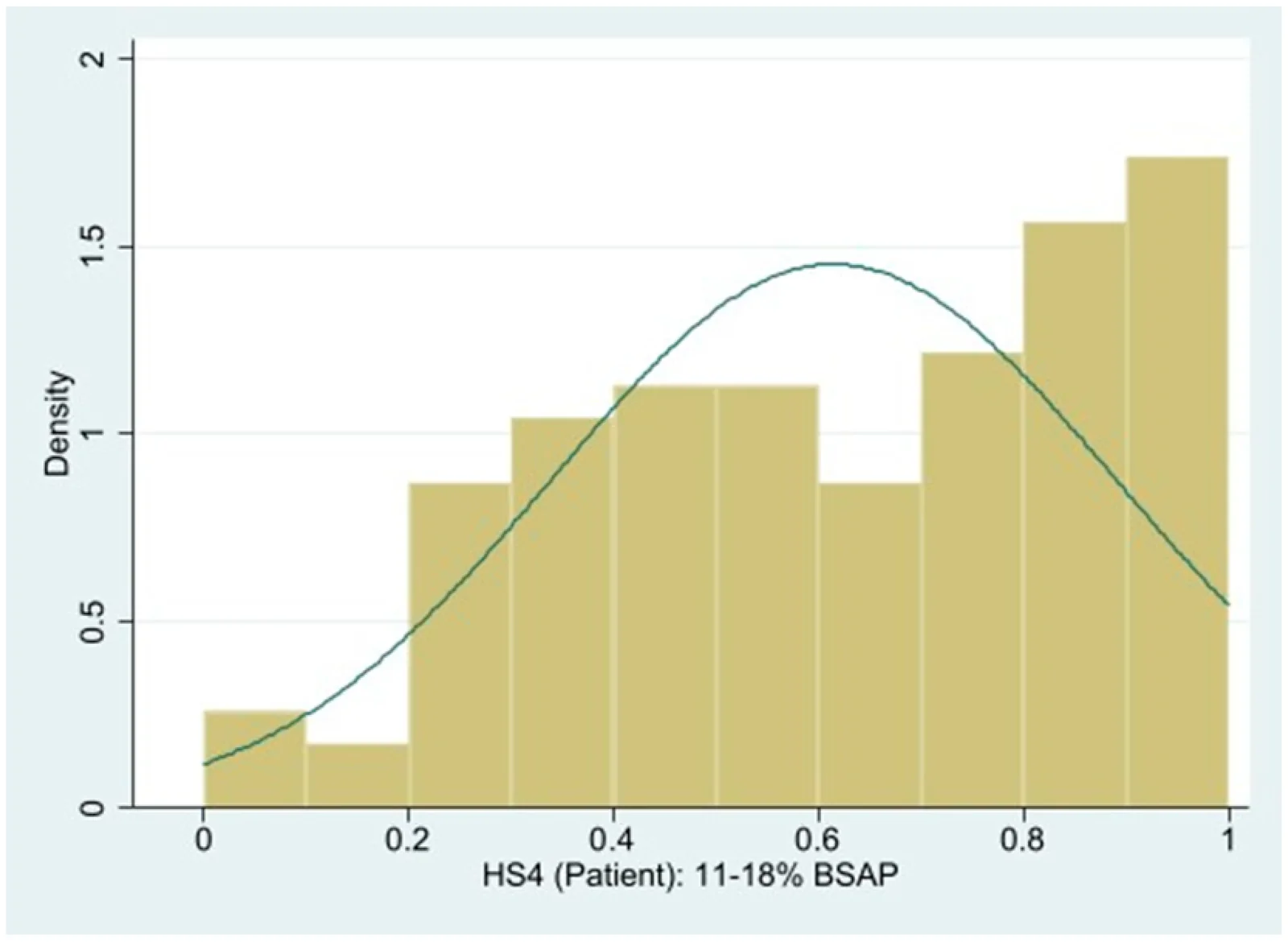
Health State 4.

**Figure A14:**
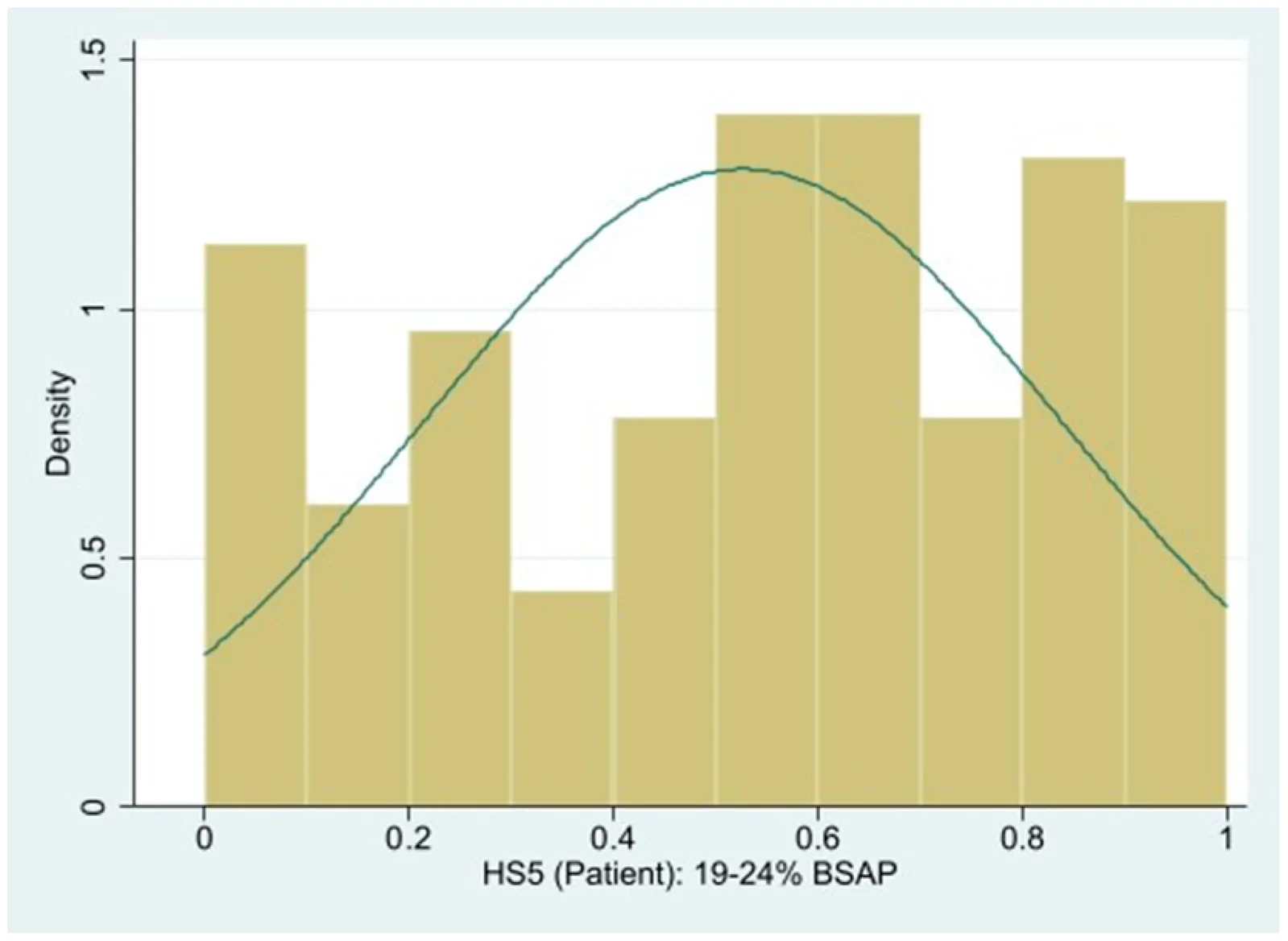
Health State 5.

**Figure A15:**
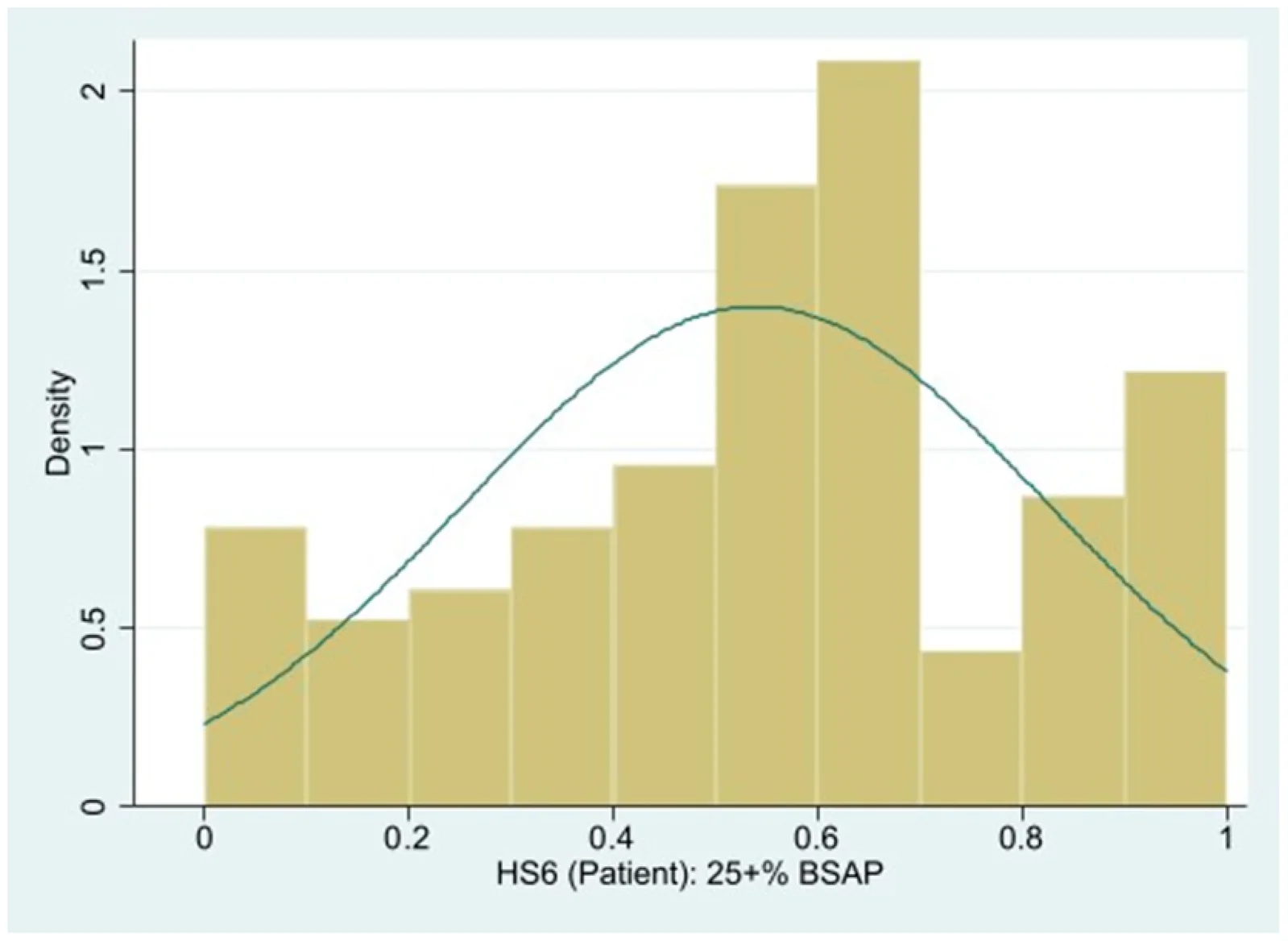
Health State 6.

**Figure A16:**
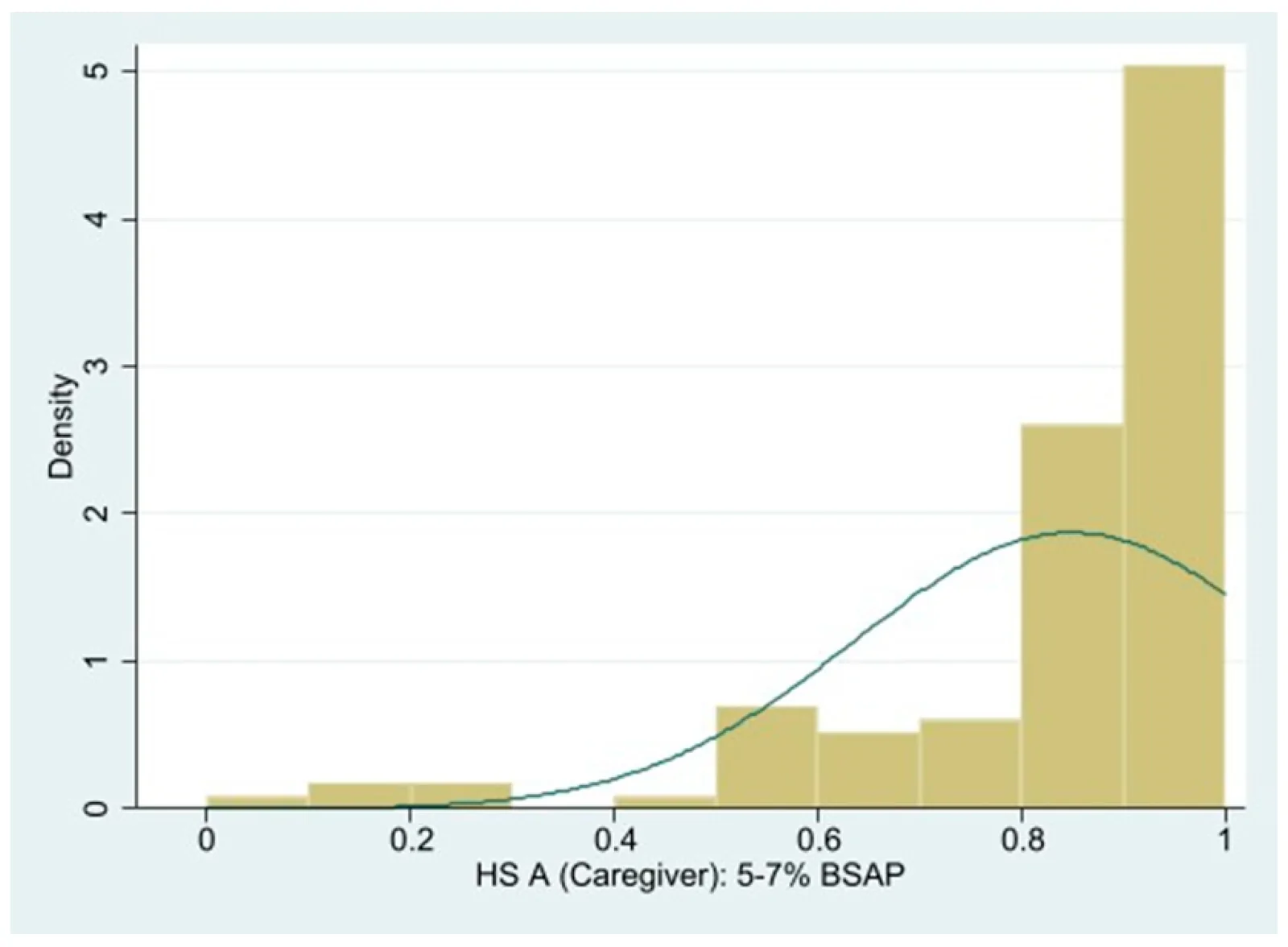
Health State A Caregiver.

**Figure A17:**
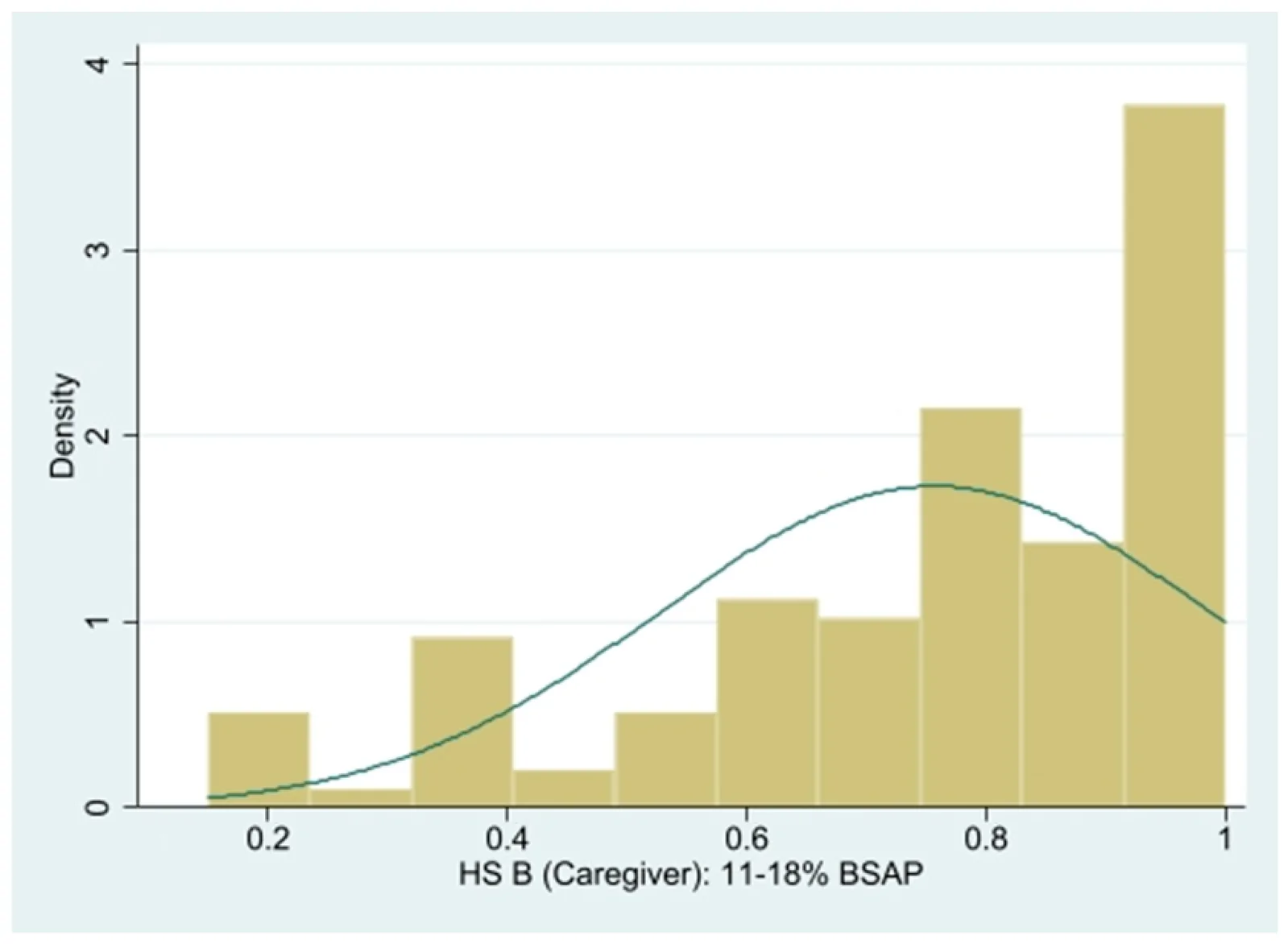
Health State B Caregiver.

**Figure A18:**
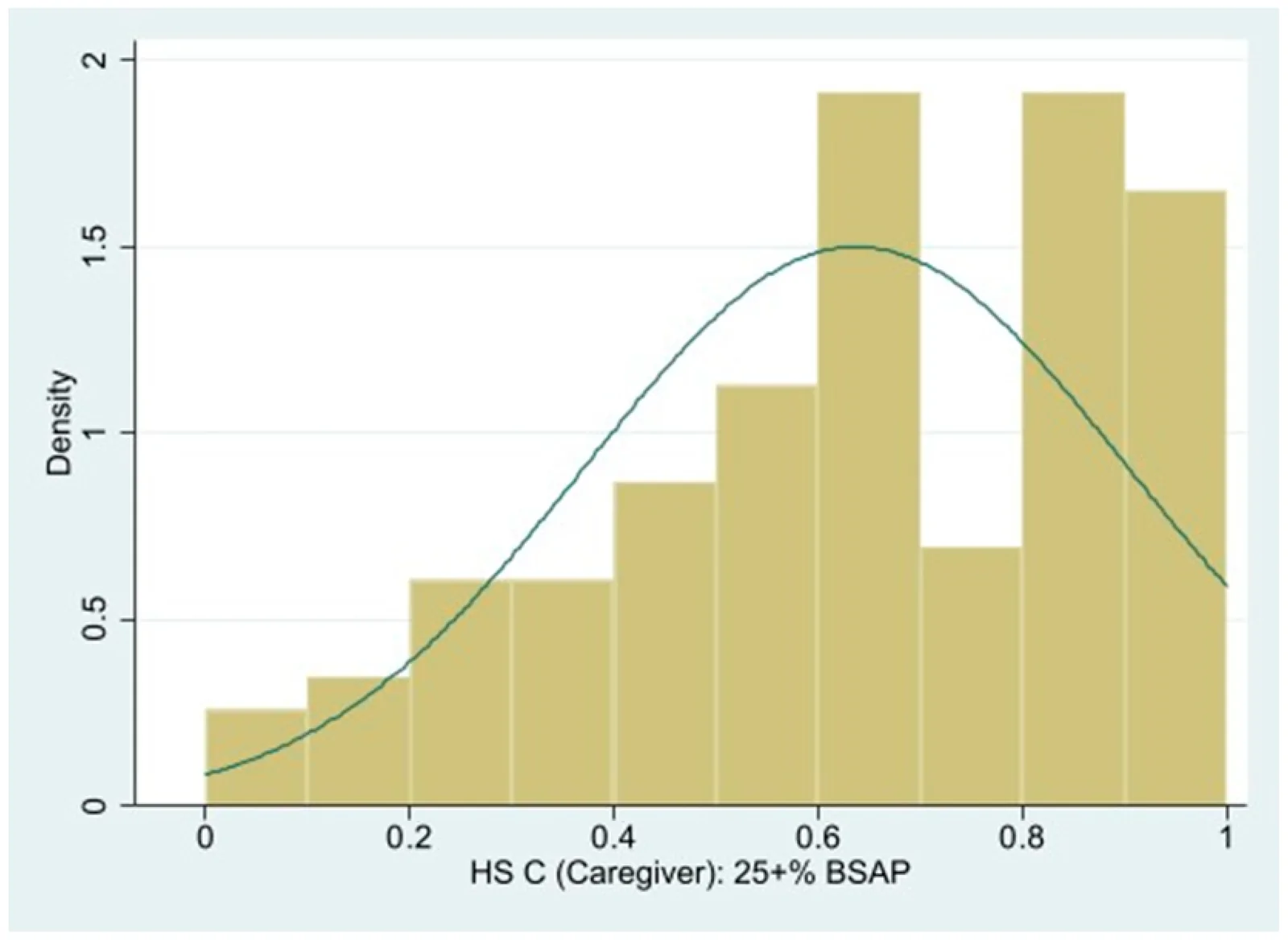
Health State C Caregiver.

## Data Availability

The data that support the findings of this study are available from the corresponding author upon reasonable request.

## Appendix A. TLR Search Strings

Epidermolysis bullosa AND (“Life quality” OR “Health-related quality of life” OR “HRQOL” OR “Quality of life” OR “Life style” OR “Karnofsky performance status” OR “Sickness impact profile” OR “Value of life” OR “Quality of life evaluation” OR “Related quality of life” OR QOLEB OR ((“surveys and questionnaires” OR (“surveys” AND “questionnaires”) OR “surveys and questionnaires” OR “questionnaire”) AND (“quality of life” OR (“quality” AND “life”) OR “quality of life”)) OR “Wellbeing” OR ((“Evaluation”] OR “Evaluation (Lond)”] OR “evaluation”) AND instrument AND (“quality of life”] OR (“quality”] AND “life”) OR “quality of life”)) OR ((“Assessment” OR “assessment”) AND instrument AND (“quality of life” OR (“quality” AND “life”) OR “quality of life”)) OR ((“Evaluation” OR “Evaluation (Lond)” OR “evaluation”) AND instrument AND (“quality of life” OR (“quality” AND “life”) OR “quality of life” OR (“life” AND “quality”) OR “life quality”)) OR ((“Assessment” OR “assessment”) AND instrument AND (“quality of life” OR (“quality” AND “life”) OR “quality of life” OR (“life” AND “quality”) OR “life quality”)) OR (“pain” OR “pain”) OR “Social relations” OR Mobility OR Functional OR (“emotions” OR “emotions” OR “emotional”)).
